# Trehalose Biosynthesis Promotes *Pseudomonas aeruginosa* Pathogenicity in Plants

**DOI:** 10.1371/journal.ppat.1003217

**Published:** 2013-03-07

**Authors:** Slavica Djonović, Jonathan M. Urbach, Eliana Drenkard, Jenifer Bush, Rhonda Feinbaum, Jonathan L. Ausubel, David Traficante, Martina Risech, Christine Kocks, Michael A. Fischbach, Gregory P. Priebe, Frederick M. Ausubel

**Affiliations:** 1 Department of Molecular Biology, Massachusetts General Hospital, Boston, Massachusetts, United States of America; 2 Department of Genetics, Harvard Medical School, Boston, Massachusetts, United States of America; 3 Department of Bioengineering and Therapeutic Sciences, University of California, San Francisco, San Francisco, California, United States of America; 4 Division of Infectious Diseases, Department of Medicine, Brigham and Women's Hospital, Boston, Massachusetts, United States of America; 5 Division of Critical Care Medicine, Department of Anesthesiology, Perioperative and Pain Medicine, Boston Children's Hospital, Boston, Massachusetts, United States of America; The University of North Carolina at Chapel Hill, United States of America

## Abstract

*Pseudomonas aeruginosa* strain PA14 is a multi-host pathogen that infects plants, nematodes, insects, and vertebrates. Many PA14 factors are required for virulence in more than one of these hosts. Noting that plants have a fundamentally different cellular architecture from animals, we sought to identify PA14 factors that are specifically required for plant pathogenesis. We show that synthesis by PA14 of the disaccharide trehalose is required for pathogenesis in Arabidopsis, but not in nematodes, insects, or mice. In-frame deletion of two closely-linked predicted trehalose biosynthetic operons, *treYZ* and *treS*, decreased growth in Arabidopsis leaves about 50 fold. Exogenously co-inoculated trehalose, ammonium, or nitrate, but not glucose, sulfate, or phosphate suppressed the phenotype of the double Δ*treYZ*Δ*treS* mutant. Exogenous trehalose or ammonium nitrate does not suppress the growth defect of the double Δ*treYZ*Δ*treS* mutant by suppressing the plant defense response. Trehalose also does not function intracellularly in *P. aeruginosa* to ameliorate a variety of stresses, but most likely functions extracellularly, because wild-type PA14 rescued the *in vivo* growth defect of the Δ*treYZ*Δ*treS* in trans. Surprisingly, the growth defect of the double Δ*treYZ*Δ*treS* double mutant was suppressed by various Arabidopsis cell wall mutants that affect xyloglucan synthesis, including an *xxt1xxt2* double mutant that completely lacks xyloglucan, even though xyloglucan mutants are not more susceptible to pathogens and respond like wild-type plants to immune elicitors. An explanation of our data is that trehalose functions to promote the acquisition of nitrogen-containing nutrients in a process that involves the xyloglucan component of the plant cell wall, thereby allowing *P. aeruginosa* to replicate in the intercellular spaces in a leaf. This work shows how *P. aeruginosa*, a multi-host opportunistic pathogen, has repurposed a highly conserved “house-keeping” anabolic pathway (trehalose biosynthesis) as a potent virulence factor that allows it to replicate in the intercellular environment of a leaf.

## Author Summary

*Pseudomonas aeruginosa* is an opportunistic human bacterial pathogen that infects a wide range of plants and animals, including the model laboratory plant *Arabidopsis thaliana*. *P. aeruginosa* utilizes many of the same virulence-related factors to infect both plants and animals. However, because plants have fundamentally different cellular architecture than animals, we hypothesized that *P. aeruginosa* synthesizes specific factors required for infecting plants but not animals. We found that synthesis of the sugar molecule trehalose, an unusual dimer of glucose, is required for plant but not animal pathogenesis. Although *P. aeruginosa* mutants defective in trehalose synthesis are non-pathogenic in Arabidopsis, Arabidopsis mutants that lack the polysaccharide xyloglucan in their cell walls can be infected by *P. aeruginosa* trehalose mutants. Moreover, application of ammonium nitrate overcomes the requirement for trehalose for infecting an Arabidopsis leaf. Our data suggest that trehalose promotes the acquisition of nitrogen-containing nutrients, thereby allowing *P. aeruginosa* to replicate in the nutrient-poor intercellular spaces in a leaf. This work shows how an opportunistic pathogen has repurposed a highly conserved “house-keeping” function (trehalose biosynthesis) as a potent virulence factor.

## Introduction

The ubiquitous bacterium *Pseudomonas aeruginosa* is a Gram-negative opportunistic pathogen that infects a wide diversity of hosts. For example, *P. aeruginosa* strain PA14 is infectious in several model genetic hosts including the plant *Arabidopsis thaliana* [1], the insect *Drosophila melanogaster* [2], and the nematode *Caenorhabditis elegans* [3]. Using these model hosts, we and others have sought to identify PA14 virulence-related factors that play key roles in pathogenesis with the goal of elucidating conserved mechanisms underlying the pathogenic process and to determine whether the spectrum of virulence-related genes in a multi-host opportunistic pathogen are distinct from the virulence genes in more specialized pathogens [1]–[7]. Our work to date suggests that PA14 virulence depends primarily on genes that are part of a conserved *P. aeruginosa* genome [8] rather than on an arsenal of host-specific virulence-related factors [8], [9].

One of the most unusual and unexpected features of *P. aeruginosa* is its ability to infect both plants and animals. Because plant cells are distinguished from metazoan cells primarily by their rigid and tough cellulosic walls, we reasoned that *P. aeruginosa* pathogenesis in plants may rely on plant-specific virulence factors related to the plant cell walls. Presumably as a consequence of these tough plant cell walls, most bacterial foliar pathogens replicate extracellularly in intercellular spaces and subvert plant cellular processes such as sugar transporters to obtain nutrients from mesophyll cells rather than attempting to directly breech plant cell walls. For example, *Xanthomonas oryzae* pv *oryzae* utilizes the Type III secretion system to inject transcriptional activators into plant mesophyll cells that upregulate the expression of sugar transporters that are not normally expressed in these cells [10].

In this paper, we report that the non-reducing disaccharide trehalose, made of two glucose residues joined by an atypical α,α-1,1-glucoside linkage, is a key virulence factor for *P. aeruginosa* PA14 pathogenesis in Arabidopsis leaves, but is not required for virulence in nematodes, flies, or mice. Trehalose is a common metabolite that has been shown to be involved in conferring tolerance to a variety of environmental stresses in diverse prokaryotic and eukaryotic species [17], [25], [26]. In PA14, trehalose is synthesized by enzymes encoded in two adjacent predicted operons, *treYZ* and *treS*, that utilize distinct mechanisms of synthesis. Deletion of these trehalose biosynthetic genes results in a highly attenuated non-pathogenic phenotype that can be rescued by trehalose and by various ammonium and nitrate sources, but not by sucrose or glucose. In addition, Arabidopsis mutants defective in the synthesis of the cell wall polymer xyloglucan also suppress the non-pathogenic phenotype of *P. aeruginosa* trehalose mutants. These data suggest that trehalose promotes the acquisition of nitrogen-containing nutrients and that the xyloglucan component of the plant cell wall is involved in this process, thereby allowing *P. aeruginosa* to replicate in the nutrient-poor intercellular spaces in a leaf. Our data show how pathogens can utilize what are normally considered to be “house-keeping” functions, such as the wide-spread ability to biosynthesize trehalose, as a potent virulence factor that allows them to replicate in the particular environment of a host.

## Results

### Trehalose production by PA14 is required for virulence in Arabidopsis

Reasoning that the tough cellulosic walls of plant cells may pose a unique challenge to plant pathogens, we surveyed the fully sequenced and annotated *P. aeruginosa* PA14 genome [4] to determine whether canonical cell wall degrading enzymes including cellulases, xylanases, and pectinases are encoded in the genome. In susceptible ecotypes (wild accessions) of Arabidopsis, *P. aeruginosa* PA14 causes soft-rot symptoms [1], typically caused by pathogens that secrete pectinases and other hydrolytic cell wall degrading enzymes. Moreover, PA14 infection causes extensive degradation of Arabidopsis mesophyll cell walls including the generation of “holes” approximately the diameter of *P. aeruginosa* through which the bacteria enter host cells [11]. We thus expected that the PA14 genome would encode a variety of cell wall degrading enzymes (CWDEs). However, our survey of the PA14 genome identified only a single, candidate cellulase, identified ambiguously as “cellulase/peptidase” (PA14_36500). Although PA14_36500 was upregulated two and three days post-inoculation *in planta*, correlating with the development of disease symptoms (Figure S1A), a transposon insertion in PA14_36500 (PA14_36500::*MAR2xT7*), in-frame deletion of the cellulose/peptidase gene (Δ*PA14_36500*), or in-frame deletion of a putative cellulase/peptidase operon (Δ*PA14_36480-36520*) did not cause a significant attenuation in virulence in Arabidopsis leaves (Table S1).

Because PA14_36500, which encodes the putative cellulose/peptidase, was induced during plant infection and because genes are often functionally clustered on bacterial genomes, we sought to identify genes adjacent to PA14_36500 that are co-regulated with PA14_36500. This led to the identification of a set of 38 genes (42.23 kb region; PA14_36375 to PA14_36830) spanning the cellulase/peptidase gene that is coordinately down-regulated in an *mvfR* (multiple virulence factor regulator) mutant grown under various culture conditions [12], [13]. Importantly, the quorum sensing-associated transcriptional regulator MvfR is required for maximum PA14 virulence in Arabidopsis [7]. Consistent with the *in vitro* transcriptional profiling data, cellulase/peptidase PA14_36500 expression was significantly reduced *in planta* in an *mvfR* mutant (Figure S1B).

**Figure 1:**
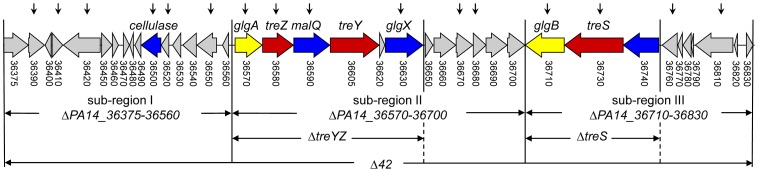
Annotation of a 42.23 kb region of the *P. aeruginosa* PA14 genome encoding 38 genes (PA14_36375–36830) and schematic representation of transposon and deletion mutants used in this study. Colors depict trehalose biosynthetic genes (red), glucanolytic genes (blue) and glucan synthesis genes (yellow). Numbers below the genes correspond to PA14 gene locus tags (http://ausubellab.mgh.harvard.edu/pa14sequencing). On the top of the Figure, vertical arrows indicate the positions of *MAR2xT7* transposon insertions [16], and on the bottom, horizontal arrows denote the extent of in-frame deletion mutants.

Besides the putative cellulase/peptidase, the PA14_36375–36830 42.23 kb region encodes putative glucanolytic enzymes (PA14_36590, PA14_36630, PA14_36740) as well as two closely linked predicted operons (http://www.pseudomonas.com), PA14_36570-36630 consisting of six genes, and PA14_36710-37640 consisting of three genes, referred to hereafter as the “*treYZ”* and “*treS”* operons, respectively, that encode enzymes involved in two different trehalose biosynthetic pathways (Figure 1; Table S2). TreY and TreZ convert maltodextrins into trehalose in a two-step enzymatic reaction [14], whereas TreS catalyzes conversion of maltose into trehalose in a single reaction [15] (Figure S2). In addition to *treY* (PA14_36605) and *treZ* (PA14_36580), the predicted *treYZ* operon contains *glgA* (PA14_36570), *malQ* (PA14_36590), hypothetical gene (PA14_36620) and *glgX* (PA14_36630). *glgA*, *malQ*, *glgX* encode enzymes with a putative role in α-1,4-linked glucan synthesis (*glgA*) and degradation (*malQ*, *glgX*), that could serve as precursors for trehalose synthesis. In addition to *treS*, the *treS* operon contains a predicted α-amylase (PA14_36740), and *glgB* (PA14_36710), a predicted α-1,4-branching enzyme (Figure 1).

The 42.23 kb PA14_36375–36830 region containing 38 genes is highly conserved among several sequenced *P. aeruginosa* strains that were examined and the *treYZ* and *treS* operons are conserved among pseudomonads in general (Table S2).

We utilized a previously constructed non-redundant PA14 transposon insertion mutant library [16] to determine whether particular PA14 genes in the 38-gene region promote pathogenesis in Arabidopsis. Among 16 transposon insertions in 16 different genes that were available in the library, two were significantly attenuated in virulence. These mutants, with insertions in *glgA* and *treZ*, exhibited a decrease in virulence of 20 and 16 fold, respectively, as measured by *in planta* growth (Table S1). *glgA* and *treZ* are the first two genes in the *treYZ* operon, pointing to an important role for trehalose in the infectious process.

**Figure 2:**
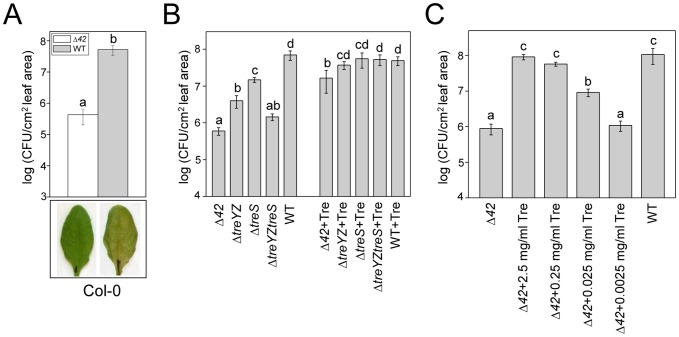
Attenuation of Δ*42* and trehalose biosynthetic mutants in Arabidopsis leaves and suppression of attenuation of trehalose mutants with exogenous trehalose. (A) Columbia (Col-0) ecotype plants were infiltrated with PA14 wild-type or Δ*42*. Leaves were harvested 3 days after infiltration and bacterial counts determined as described in Materials and Methods. Representative photographs of infected leaves were taken 3 days after infiltration. Four independently constructed Δ*42* mutants exhibited the same dramatic non-pathogenic phenotype in Arabidopsis leaves. The experiment was repeated more than three times. (B, C) Before the infiltration of leaves with PA14 wild-type or PA14-derived deletion mutants, trehalose (Tre) at the indicated concentrations was added to bacterial suspensions. In panel B, the trehalose concentration was 2.5 mg/ml. The leaves were harvested 3 days post-infiltration (dpi) and bacterial counts determined as described in Materials and Methods. Data represent the mean of bacterial titers ± SE of six leaf disks excised from 6 leaves of 3 plants. Letters above bars denote statistically significant differences (P<0.05, Fisher's PLSD test). See Figure 1 for a description of the mutants. The experiments in (B) and (C) were repeated four times and two times, respectively.

To further investigate whether the trehalose operons and/or other genes in the 38-gene cluster are required for virulence, we constructed an in-frame deletion of the entire 42.23 kb region (referred to hereafter as Δ*42*) by homologous recombination. In contrast to insertions in *glgA* and *treZ*, which exhibited at most a 20 fold decrease in growth compared to wild-type, the Δ*42* mutant exhibited severe attenuation in virulence, affecting growth of PA14 infiltrated into Arabidopsis leaves about 120 fold and preventing the appearance of pathogenic symptoms (Figure 2A). Similar results were obtained with four independently constructed Δ*42* mutants (data not shown), demonstrating that the non-pathogenic phenotype was caused by the deletion of the 42.23 kb region. Importantly, the Δ*42* mutant does not appear to be slow growing or to be generally deficient in a variety of phenotypes associated with virulence in *P. aeruginosa*. The Δ*42* deletion mutant was not auxotrophic, grew at the same rate as wild-type PA14 in a variety of minimal and rich media, and had no observable phenotypes with respect to the production of pyocyanin (Figure S3), motility, or biofilm formation (Table S3), and similar results were obtained with a second independently-constructed Δ*42* mutant (Figure S3; Table S3). Because independently-constructed Δ*42* mutants exhibited the same phenotypes, one of the Δ*42* mutants was chosen for subsequent experiments.

We next constructed several smaller deletions within the 42 kb region to determine which of the 38 encoded genes are primarily responsible for the severe avirulent phenotype of Δ*42*: Δ*PA14_36375-36560* (sub-region I) contains a deletion of the cellulase/peptidase operon and several adjacent genes, and Δ*PA14_36570-36700* (sub-region II) and Δ*PA14_36710-36830* (sub-region III) contain deletions of the *treYZ* and *treS* genes, respectively, including some neighboring genes (Figure 1).

Deletion of sub-region I that includes the putative cellulase/peptidase gene had a modest 3.3 fold reduction in virulence. In contrast, deletion of sub-region II that contains the *treYZ* operon had a much more significant effect on virulence (28.7 fold decrease in growth; Figure S4), whereas deletion of sub-region III that contains the *treS* operon caused a 5.9 fold decrease in growth (Figure S4). These experiments suggested that the *treYZ* and *treS* operons play a significant role in PA14 pathogenesis in Arabidopsis.

To corroborate the involvement of the trehalose genes in plant pathogenesis we constructed Δ*PA14_36570-36630* (Δ*treYZ*) and Δ*PA14_36710-36740* (Δ*treS*) containing deletions of only the two putative operons containing the *treYZ* and *treS* genes, respectively, and Δ*PA14_36570-36630*;*PA14_36710-36740* (Δ*treYZ*Δ*treS*) containing deletions of both of the trehalose biosynthetic operons (Figure 1). Deleting either the putative *treYZ* or the *treS* operons (Figure 2B) had approximately the same effects as deleting the more extensive corresponding subregions II or III, respectively (Figure S4), and deleting both trehalose operons resulted in an approximately 50 fold decrease in virulence compared to the approximate 120 fold decrease observed with the Δ*42* mutant (Figure 2B). These data show that the *treYZ* and *treS* operons play a key role in pathogenesis in Arabidopsis leaves, but that genes in the 42 kb region in addition to those involved in trehalose biosynthesis also play a role in plant pathogenesis.

Further evidence suggesting an important role for trehalose biosynthesis in plant pathogenesis was obtained by measuring the levels of trehalose synthesized *in vitro* by PA14 wild-type and trehalose biosynthetic mutants. While wild-type PA14 synthesized readily detectable levels of trehalose, there was approximately 50% less trehalose in the *ΔtreS* mutant, and there were undetectable levels of trehalose in the *glgA*, *treZ*, Δ*treYZ*, Δ*treYZ*Δ*treS*, and Δ*42* mutants (Table 1). These data show that the *treYZ* and *treS* operons encode enzymes involved in trehalose biosynthesis. These data also suggest that *treS* operon may be dependent on *treYZ* for trehalose production, as reported previously [17]. When we compared the levels of trehalose synthesized *in vitro* (Table 1) and the extent of growth of the various strains in Arabidopsis leaves (Figure 2B; Table S1), we found an excellent positive correlation coefficient (R^2^ = 0.87).

**Table 1:** Trehalose levels in the trehalose mutants.

Strains	Trehalose (mg/ml)
1. PA14_36570::MAR2xT7 (*glgA*)	not detected
2. PA14_36580::MAR2xT7 (*treZ*)	not detected
3. Δ*PA14_36570-36630* (Δ*treYZ*)	not detected
4. Δ*PA14_36710-36740* (Δ*treS*)	0.212±0.015
5. Δ*PA14_36570-36630*;*PA14_36710-36740* (Δ*treYZtreS*)	not detected
6. Δ*PA14_36375-36830* (Δ*42*)	not detected
7. WT	0.444±0.003

*P. aeruginosa* strains were grown at 37°C in MinA medium supplemented with 0.5 M NaCl. Trehalose was extracted and quantified enzymatically as described in Materials and Methods. See Figure 1 for a description of the mutants. Data represent the mean ± SE of two replicate samples and are representative of at least three independent experiments.

Importantly, we found that co-infiltration of the PA14 trehalose mutants and pure trehalose essentially completely suppressed the avirulent phenotypes of the Δ*treYZ, ΔtreS*, and Δ*treYZΔtreS* mutants and mostly suppressed the phenotype of the Δ*42* mutant (Figures 2B and 2C). However, 0.25 mg/ml trehalose also rescued the Δ*42* mutant almost as well as 2.5 mg/ml, and 0.025 mg/ml trehalose partially suppressed the growth defect of the Δ*42* mutant (Figure 2C). These data indicated a requirement for trehalose for PA14 virulence *in planta*, potentially at physiologically relevant concentrations.

In summary, the data in this section shows that the Δ*treYZ, ΔtreS*, and Δ*treYZΔtreS* mutants are less virulent *in planta*, that they either synthesize undetectable (Δ*treYZ* and Δ*treYZΔtreS*) or reduced (*ΔtreS*) levels of trehalose, that their level of virulence positively correlates with the level of trehalose they synthesize, and that their reduced virulence phenotype can be suppressed by exogenous trehalose. These data demonstrate that the virulence deficient phenotypes of the Δ*treYZ*, *ΔtreS*, and Δ*treYZΔtreS* mutants are a consequence of the inability of these strains to synthesize trehalose, thereby correlating the genotype of these mutants with their avirulent phenotypes.

### Trehalose biosynthetic genes are not required for virulence in metazoans

**Figure 3:**
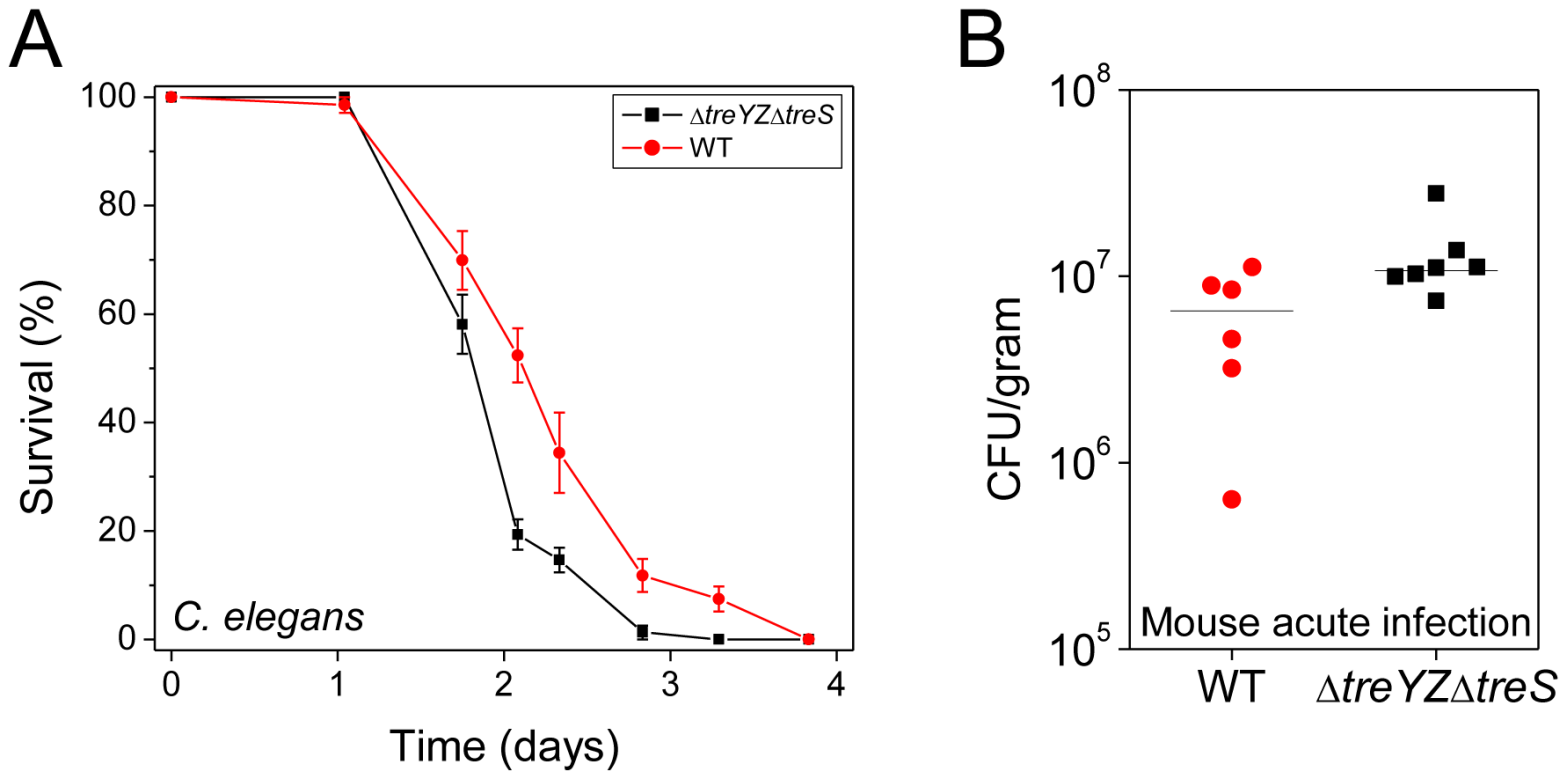
The *ΔtreYZΔtreS* mutant is more virulent than wild-type PA14 in nematodes and mice. (A) *C. elegans* are more susceptible to killing by *ΔtreYZΔtreS* than PA14 wild-type (P<0.0001). Mutant *fer15;fem1 C. elegans* animals were exposed to *P. aeruginosa* strains and survival was determined as described in Materials and Methods. Data at each time point correspond to the average of three plates per strain, each with approximately 40 animals per plate, and are representative of two independent experiments. (B) The Δ*42* mutant is more virulent than wild-type PA14 in a murine acute lung infection model. See Materials and Methods for details of infection protocol. The median CFU/gram of lung tissue of mice infected with *ΔtreYZΔtreS* is 2-fold higher than with wild-type PA14 18 hours post intranasal infection (P<0.05, Mann-Whitney U test). Data are representative of two independent experiments.

As described in the Introduction, PA14 infection models have previously been established in *C. elegans* [3], *D. melanogaster* [18], and mice [19], [20], as well as in other metazoans. Interestingly, the Δ*treYZΔtreS* double trehalose mutant was not less virulent in a *C. elegans* killing model or in a murine acute pneumonia model. In fact, the Δ*treYZΔtreS* appeared to be slightly more virulent in the metazoan hosts (Figure 3). Similar results were obtained with the Δ*42* mutant in these two models as well as in a *D. melanogaster* ingestion model and in a chronic oropharyngeal colonization model in transgenic mutant mice lacking the cystic fibrosis transmembrane conductance regulator protein (see Materials and Methods for the mutant description)(Figure S5). These data suggest that trehalose appears to be specifically required for plant but not for metazoan pathogenesis. In the sections that follow, we considered several hypotheses concerning the role of trehalose in promoting the virulence of *P. aeruginosa* during the infectious process in plants but not in animals.

### Specific Arabidopsis cell wall mutants suppress the phenotype of PA14 trehalose mutants

Since a major difference between plant and animals cells is the plant cellulosic cell wall, we reasoned that trehalose may function in a process that involves the plant cell wall. Because PA14 infection in Arabidopsis leaves causes extensive degradation of mesophyll cell walls [11], we first investigated the possibility that trehalose enhances the activity of cell wall degrading enzymes (CWDEs). We tested whether trehalose enhanced the activity of a variety of commercial CWDEs to hydrolyze partially purified Arabidopsis cell walls *in vitro* to generate reducing sugars, which were measured using the Somogyi-Nelson assay [21], [22]. However, we were not able to conclusively demonstrate that trehalose enhanced the activity of the CWDEs tested (data not shown).

**Figure 4:**
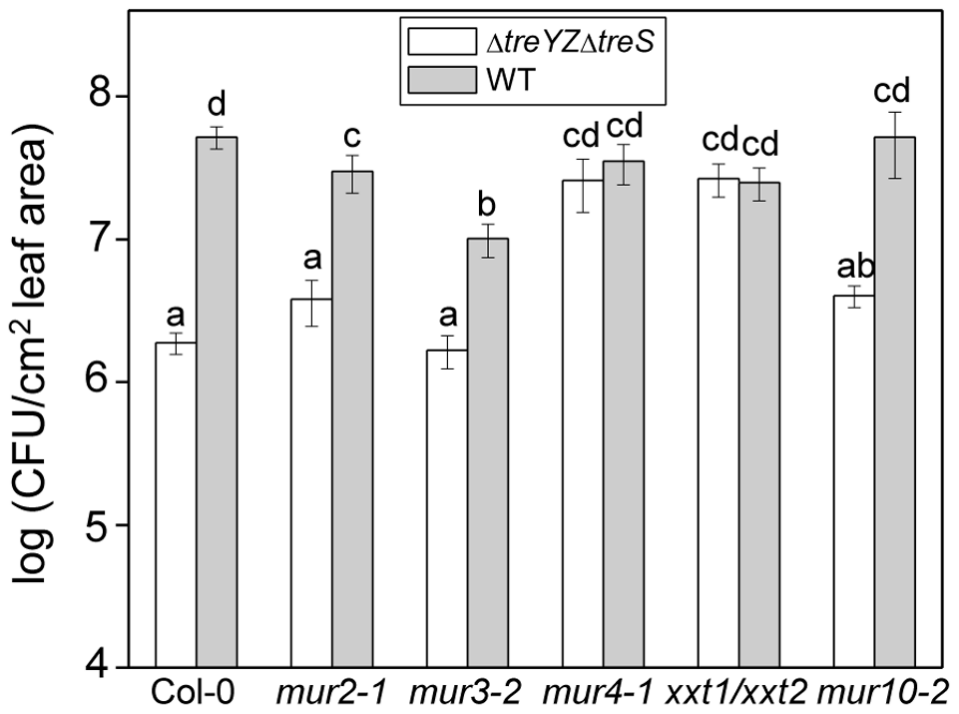
The *in planta* growth defect of the PA14 *ΔtreYZΔtreS* mutant in Arabidopsis is suppressed by cell wall mutations. Growth of PA14 wild-type and the *ΔtreYZΔtreS* mutant 3 days post infiltration in Arabidopsis cell wall mutants *mur2-1*, *mur3-2*, *mur4-1*, *mur10-2* and *xxt1/xxt2*. Data represent the mean of bacterial titers ± SE of six leaf disks excised from 6 leaves of 3 plants. Letters above bars denote statistically significant differences (P<0.05, Fisher's PLSD test). The experiments were repeated at least two times.

We next reasoned that if trehalose interacts with the plant cell wall, specific Arabidopsis cell wall mutants might suppress the phenotype of the Δ*treYZΔtreS* mutant. We tested the growth of wild-type PA14 and the Δ*treYZΔtreS* mutant in several Arabidopsis cell wall mutants involved in xyloglucan (*mur2-1*, *mur3-2*, *xxt1/xxt2*), arabinose (*mur4-1*), or cellulose (*mur10-2*) synthesis. Remarkably, the Δ*treYZΔtreS* mutant grew to the same titer as wild-type PA14 in an *xxt1/xxt2* double mutant that completely lacks xyloglucan in its cell walls and in a *mur4-1* mutant that has decreased levels of arabinose in pectins, xylans, and xyloglucans [23] (Figure 4). Similar results were obtained with the Δ*42* mutant; i.e., the Arabidopsis *xxt1/xxt2* mutant completely suppressed and the *mur4-1* mutant mostly suppressed the avirulent phenotype of the Δ*42* mutant (Figure S6).

**Figure 5:**
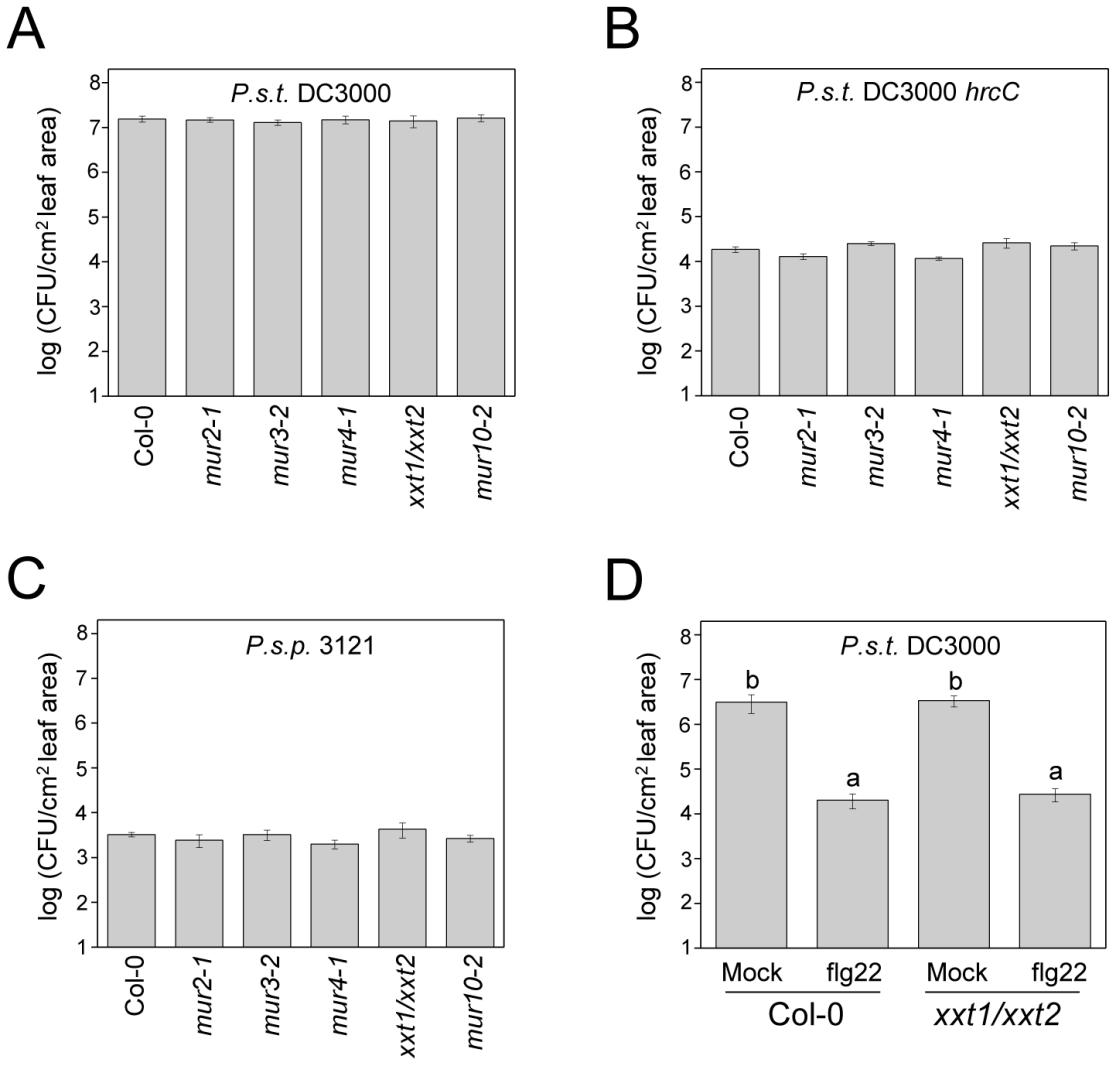
Arabidopsis cell wall mutants are not more susceptible to virulent or non-pathogenic *P. syringae* strains and the *xxt1/xxt2* mutant mounts an effective innate immune response. Growth of (A) *P. syringae* pv. *tomato* strain DC3000 (B) *P. syringae* pv. *tomato* strain DC3000 *hrcC* (C) *P. syringae* pv. *phaseolicola* strain 3121 three days post infiltration in Arabidopsis cell wall mutants *mur2-1*, *mur3-2*, *mur4-1*, *mur10-2* and *xxt1/xxt2* and Col-0 wild-type and (D) *P. syringae* pv. *tomato* strain DC3000 three days post infiltration of Col-0 and *xxt1/xxt2* mutant preinfiltrated with 1 µM flg22 for 24 hours. Data represent the mean of bacterial titers ± SE of six leaf disks excised from 6 leaves of 3 plants. Letters above bars denote statistically significant differences (P<0.05, Fisher's PLSD test). Absence of letters indicates no statistically significant differences. The experiments were repeated at least two times.

We ruled out the possibility that the Arabidopsis cell wall mutants suppress the avirulent phenotype of the PA14 trehalose mutants simply because they are generally more susceptible to pathogen attack. As shown in Figure 5A, the cell wall mutants did not exhibit enhanced susceptibility to the *P. syringae* pv. *tomato* strain DC3000, a well-studied *bona fide* Arabidopsis pathogen. The Arabidopsis cell wall mutants were also not more susceptible to a DC3000 *hrcC* mutant (Figure 5B), which is greatly impaired in virulence, or to the bean pathogen *P. syringae* pv. *phaseolicola* strain 3121 (Figure 5C), which is not normally pathogenic in Arabidopsis. Consistent with these data, we also showed that the *xxt1/xxt2* mutant, which exhibits the most severe cell wall defect of the Arabidopsis mutants tested, mounts a normal defense response when challenged with the flagellin peptide flg22 (Figure 5D). Flg22 elicits so-called “pattern triggered immunity” in Arabidopsis. When Arabidopsis leaves are pre-infiltrated with flg22, flg22 exerts a protective effect against subsequent infection with *P. syringae* DC3000 [24]. As shown in Figure 5D, flg22 elicits the same level of protection against *P. syringae* DC3000 in *xxt1xxt2* plants as in wild-type plants.

### Ammonium and nitrate but not glucose or sucrose suppress the phenotype of trehalose mutants

As described in the Introduction, because bacterial plant pathogens primarily replicate in the intercellular spaces in a leaf, they need to acquire nutrients from plant mesophyll cells. We therefore tested whether trehalose may be involved in the acquisition of a variety of nutrient sources including carbon, nitrogen, sulfur and phosphorous. If this were the case, we reasoned that co-infiltration of particular nutrients with the Δ*treYZΔtreS* or the Δ*42* mutant would suppress their non-pathogenic phenotypes.

**Figure 6:**
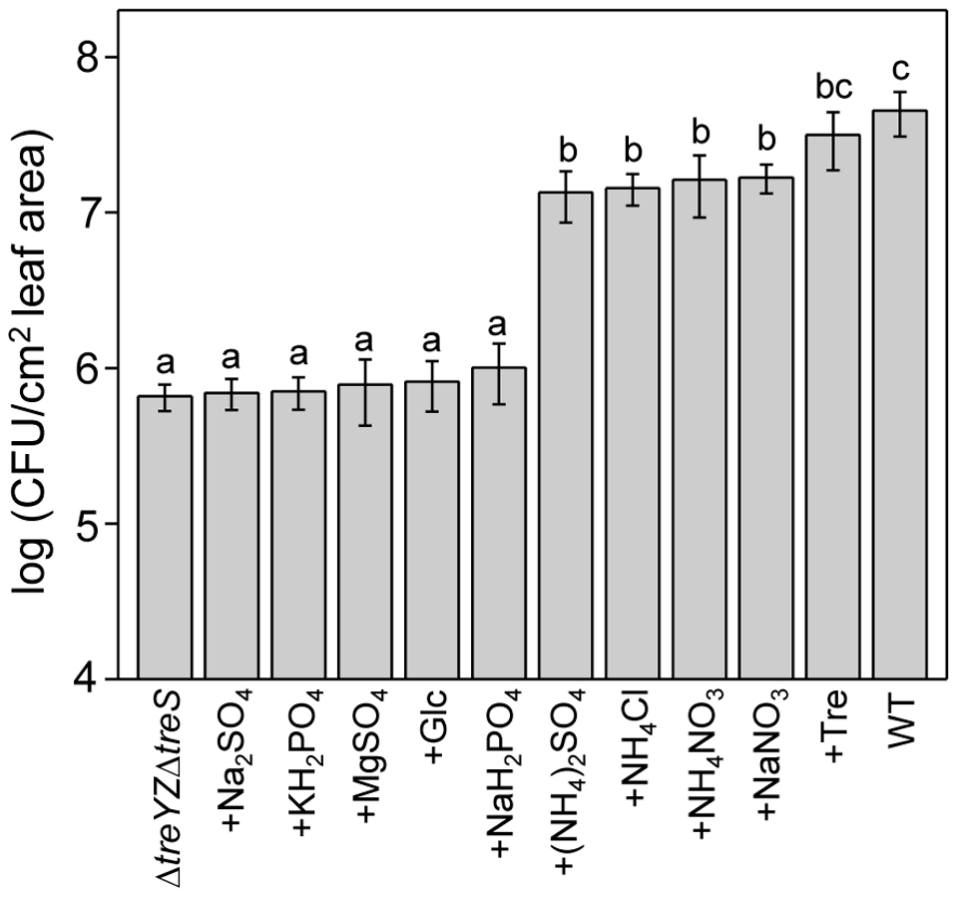
The *in planta* growth defect of the *ΔtreYZΔtreS* double mutant is suppressed by ammonium or nitrate ions. Leaves of four-week-old Arabidopsis Col-0 plants were infiltrated with PA14 wild-type or with Δ*42* co-inoculated with various solutions of phosphate, sulfate, nitrate, or ammonium salts at 1 mM. Suppression of the growth defect of Δ*42* with 2.5 mg/ml trehalose (Tre) and 1.25 mg/ml glucose (Glc) were tested as positive and negative controls, respectively. Data represent the mean of bacterial titers ± SE of six leaf disks excised from 6 leaves of 3 plants. Letters above bars denote statistically significant differences (P<0.05, Fisher's PLSD test). The experiments were repeated at least two times.

Co-infiltration of the Δ*treYZΔtreS* double mutant with glucose (Figure 6) or co-infiltration of the Δ*42* mutant with glucose or sucrose (Figure S7A) did not rescue the attenuated phenotype in the Arabidopsis leaf assay. These experiments showed that the Δ*treYZΔtreS* or the Δ*42* mutant is not limited by carbon. The fact that trehalose but not glucose or sucrose suppressed the phenotype of the Δ*42* mutant also shows that the putative cellulase/peptidase and other hypothetical glucanolytic enzymes encoded in the 38 gene region deleted in the Δ*42* mutant do not play a critical role in supplying a carbon source to PA14.

We also entertained the possibility that PA14 could accumulate trehalose as a storage sugar, analogous to glycogen or starch, and then hydrolyze trehalose using the enzyme trehalase (PA14_33450, *treA*) and utilize the resulting glucose as a carbon source, thereby promoting virulence. We ruled out this possibility, however, by showing that co-infiltration of a double Δ*42treA::MAR2xT7* mutant (which cannot metabolize trehalose) with trehalose rescued the non-pathogenic phenotype similarly as co-infiltration of the Δ*42* mutant with trehalose (Figure S7B). We also confirmed that the Δ*42treA::MAR2xT7* cannot metabolize trehalose and utilize it as a carbon source (see Materials and Methods).

Finally, we tested various salts to determine whether they would suppress the phenotypes of the Δ*treYZΔtreS* (Figure 6) or the Δ*42* mutant (Figure S8). Interestingly, ammonium and nitrate ions almost completely suppressed the lack of growth phenotype of the Δ*treYZΔtreS* (Figure 6) or the Δ*42* mutant (Figure S8), whereas sulfates and phosphates did not have a significant effect.

**Figure 7:**
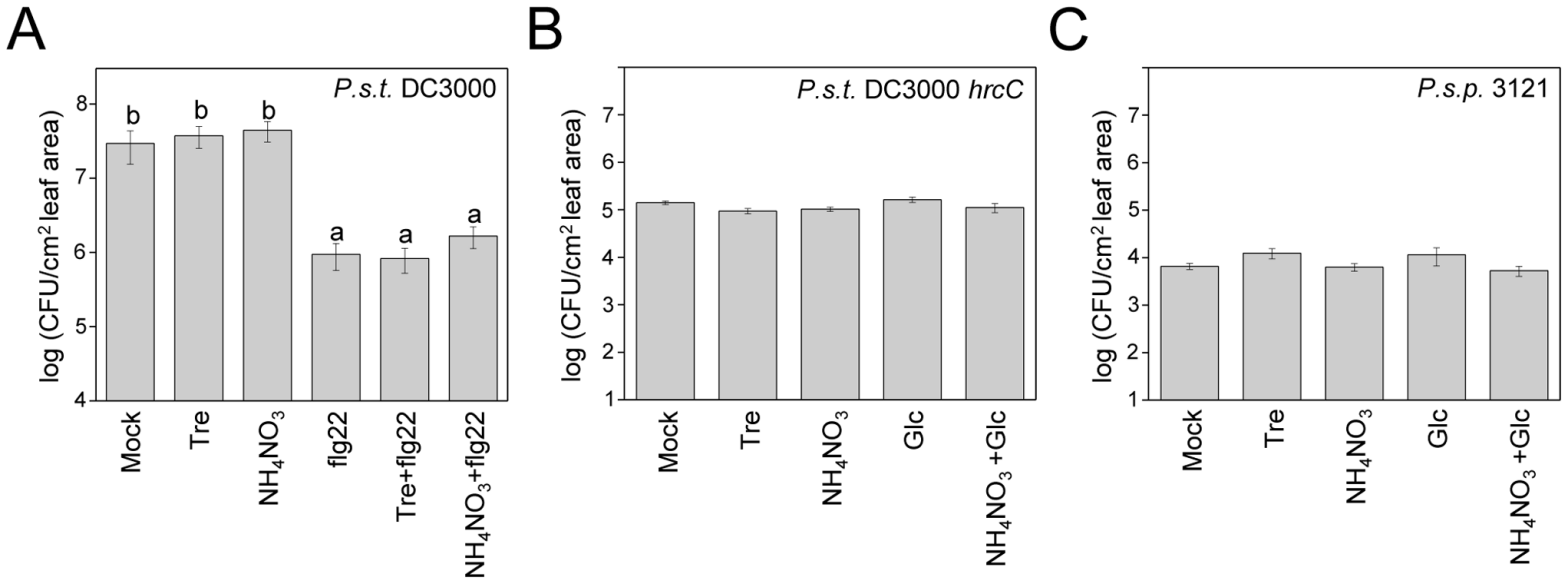
Trehalose or ammonium nitrate does not suppress the Arabidopsis flg22-mediated defense response and does not make Arabidopsis more susceptible to non-pathogenic *P. syringae* strains. (A) Growth of *P. syringae* pv. *tomato* strain DC3000 three days post infiltration of Col-0 plants pretreated for 24 hours with 1 µM flg22, 1 mM trehalose, 1 mM ammonium nitrate individually or with a mixture of flg22 with trehalose or ammonium nitrate. (B) *P. syringae* pv. *tomato* strain DC3000 *hrcC* or (C) *P. syringae* pv. *phaseolicola* strain 3121 were co-inoculated with 1 mM trehalose or 1 mM ammonium nitrate and bacterial counts determined 3 days post infiltration. Glucose or glucose plus ammonium nitrate were included as controls. Data represent the mean of bacterial titers ± SE of six leaf disks excised from 6 leaves of 3 plants. Letters above bars denote statistically significant differences (P<0.05, Fisher's PLSD test). Absence of letters indicates no statistically significant differences. The experiments were repeated at least two times.

The data in this section suggest that trehalose enhances access to nitrogen sources during an Arabidopsis infection. An alternative model is that ammonium nitrate (as well as trehalose) suppresses the avirulent phenotype of the PA14 trehalose mutants by suppressing the plant defense response. To test this possibility, we tested whether infiltration of leaves with trehalose or ammonium nitrate resulted in enhanced susceptibility to *P. syringae* DC3000 (Figure 7A), the DC3000 *hrcC* mutant (Figure 7B), or *P. syringae* pv. *phaseolicola* strain 3121 (Figure 7C); however, neither trehalose nor ammonium nitrate increased the susceptibility to any of these strains. Moreover, infiltration of trehalose or ammonium nitrate did not block the ability of flg22 to elicit protection against infection by *P. syringae* DC3000 (Figure 7A).

### Trehalose does not appear to function as a stress-response molecule either *in vivo* or *in vitro*

Trehalose is well-studied as a so-called compatible solute, which is defined as a molecule that functions as an osmolyte and helps an organism survive osmotic stress. We therefore tested whether other di- and trisaccharide compatible solutes would suppress the avirulent phenotype of the Δ*42* mutant. Indeed, as shown in Figure S9, both maltose and maltotriose functioned similarly to trehalose in allowing the Δ*42* mutant to grow *in planta*, albeit somewhat less efficiently than did trehalose.

**Figure 8:**
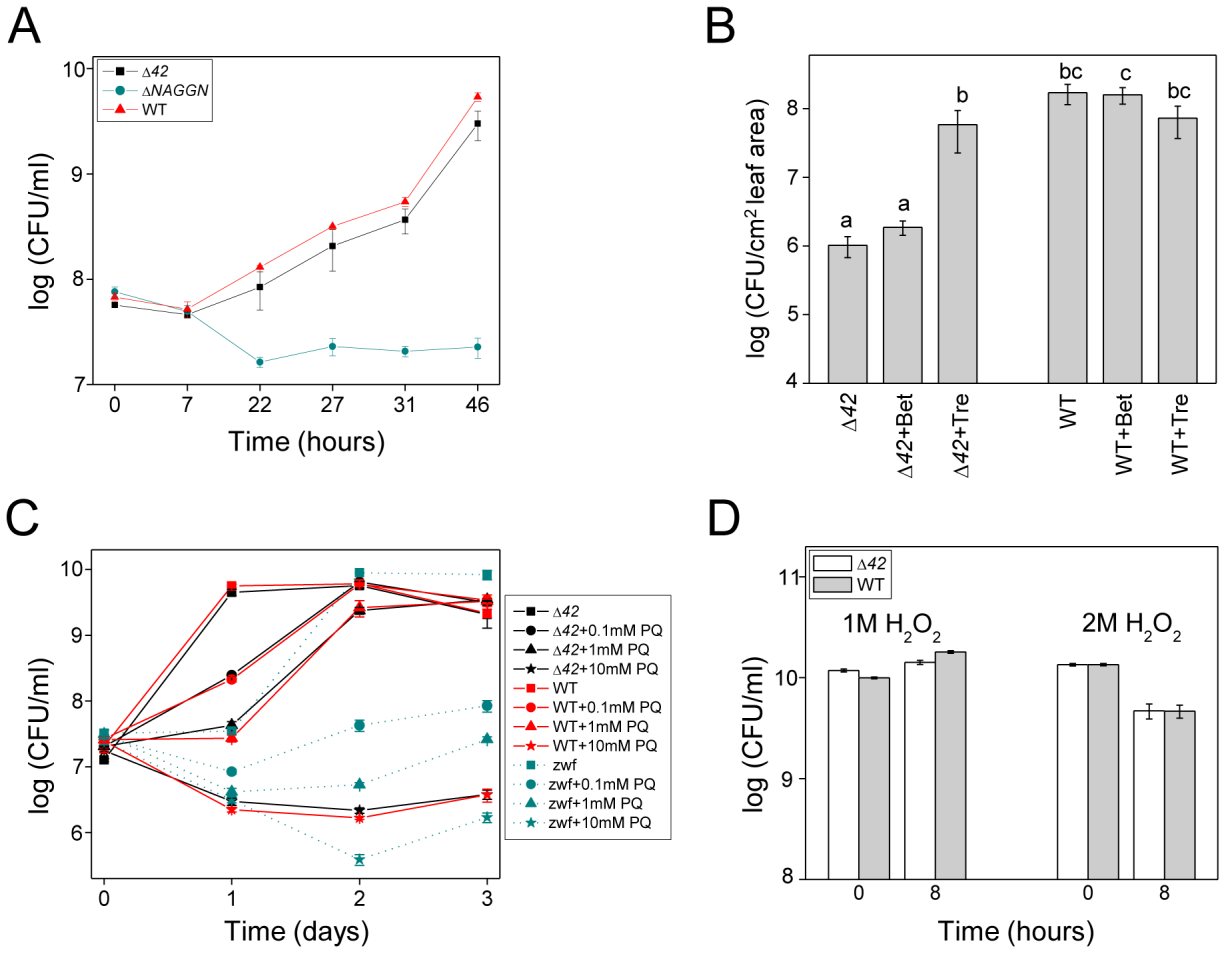
The Δ*42* mutant is not more susceptible to osmotic or oxidative stress. (A) Growth of Δ*42, ΔNAGGN* and PA14 wild-type under osmotic stress *in vitro*. Cells were grown at 37°C in MinA medium supplemented with 0.5 M NaCl. Data represent the mean ± SE of 3 replicates. (B) The *in planta* growth defect of Δ*42* is suppressed by trehalose but not betaine. See Figure 2 for experimental details. Data represent the mean ± SE of six replicate samples. (C) *In vitro* survival of PA14 wild-type, Δ*42*, and a PA14 *zwf*::*MAR2xT7* mutant cultured for three days in MinA medium supplemented with various concentrations of paraquat (PQ). (D) Survival of PA14 wild-type and Δ*42* in LB medium containing 1 M or 2 M hydrogen peroxide added directly to overnight cultures grown for 14 h (inoculum, zero time point on x axis). Cells were further incubated for 8 h at 37°C. 3 M H_2_O_2_ was a lethal dose. All experiments in Figure 8 were repeated at least two times.

Given these results, we next considered the hypothesis that trehalose enhances the virulence of PA14 by ameliorating a variety of environmental stresses [17], [25], [26]. However, the Δ*42* mutant was not more susceptible than wild-type PA14 to osmotic stress in response to 0.5 M NaCl (Figure 8A). As a positive control for the osmotic stress experiment, we constructed an in-frame deletion of a predicted (http://www.pseudomonas.com) three-gene operon (PA14_19350-19370) responsible for the synthesis of a major organic osmoprotectant in *P. aeruginosa*, *N*-acetylglutaminylglutamine amide (NAGGN) [27]. As expected, the Δ*PA14_19350-19370* mutant (Δ*NAGGN*) was more susceptible to 0.5 M NaCl than wild-type PA14 or the Δ*42* mutant (Figure 8A).

We further tested whether trehalose functions to protect PA14 from osmotic stress *in vitro* by comparing its ability to enhance growth in minimal medium supplemented with 0.5 M NaCl compared to the well-studied osmoprotectant molecule betaine [27]. *In vitro*, betaine rescued the growth of PA14, Δ*42*, and the Δ*NAGGN* mutant in 0.5 M NaCl whereas trehalose had no effect (Figure S10A). We also tested whether betaine would rescue the Δ*42* mutant for *in planta* growth, similarly to trehalose. However, as shown in Figure 8B, betaine had no significant effect in rescuing Δ*42* growth *in planta*, showing that the ability of trehalose to rescue Δ*42 in planta* is not likely due to the fact that it is functioning to protect Δ*42* from osmotic stress. In contrast to Δ*42*, the Δ*NAGGN* mutant, which is very susceptible to osmotic stress *in vitro*, had no significant impairment in growth *in planta* (Table S1). These data show that the Δ*42* mutant is not highly susceptible to osmotic stress and that trehalose does not play a major role as an osmoprotectant in PA14.

As an alternative to functioning as an osmoprotectant, we investigated whether trehalose protects PA14 from reactive oxygen-mediated stress generated as a consequence of the plant innate immune response. However, we found no significant difference between the Δ*42* mutant and wild-type PA14 with respect to tolerance to paraquat or hydrogen peroxide (Figures 8C and 8D, respectively). Because a *P. aeruginosa zwf* mutant has been reported to be hyper-sensitive to paraquat-mediated killing [28], we also tested a PA14 *zwf::MAR2xT7* mutant [16] as a positive control for determining the sensitivity of PA14 and Δ*42* to paraquat. As shown in Figure 8C, the *zwf* mutant exhibited enhanced susceptibility to paraquat *in vitro*, but did not exhibit an impaired growth phenotype *in planta* (Table S1). These data show that it is unlikely that trehalose functions to protect PA14 from oxidative stress.

In addition to oxidative and osmotic stress, we also tested whether the Δ*42* mutant is susceptible to pH or temperature stress, displayed a defect in biofilm formation under osmotic stress, or was deficient in the generation of persister cells in the presence of antibiotics. However, wild-type PA14 and the Δ*42* mutant were indistinguishable in all of these tests (Figure S10B–E).

### Trehalose functions extracellularly

**Figure 9:**
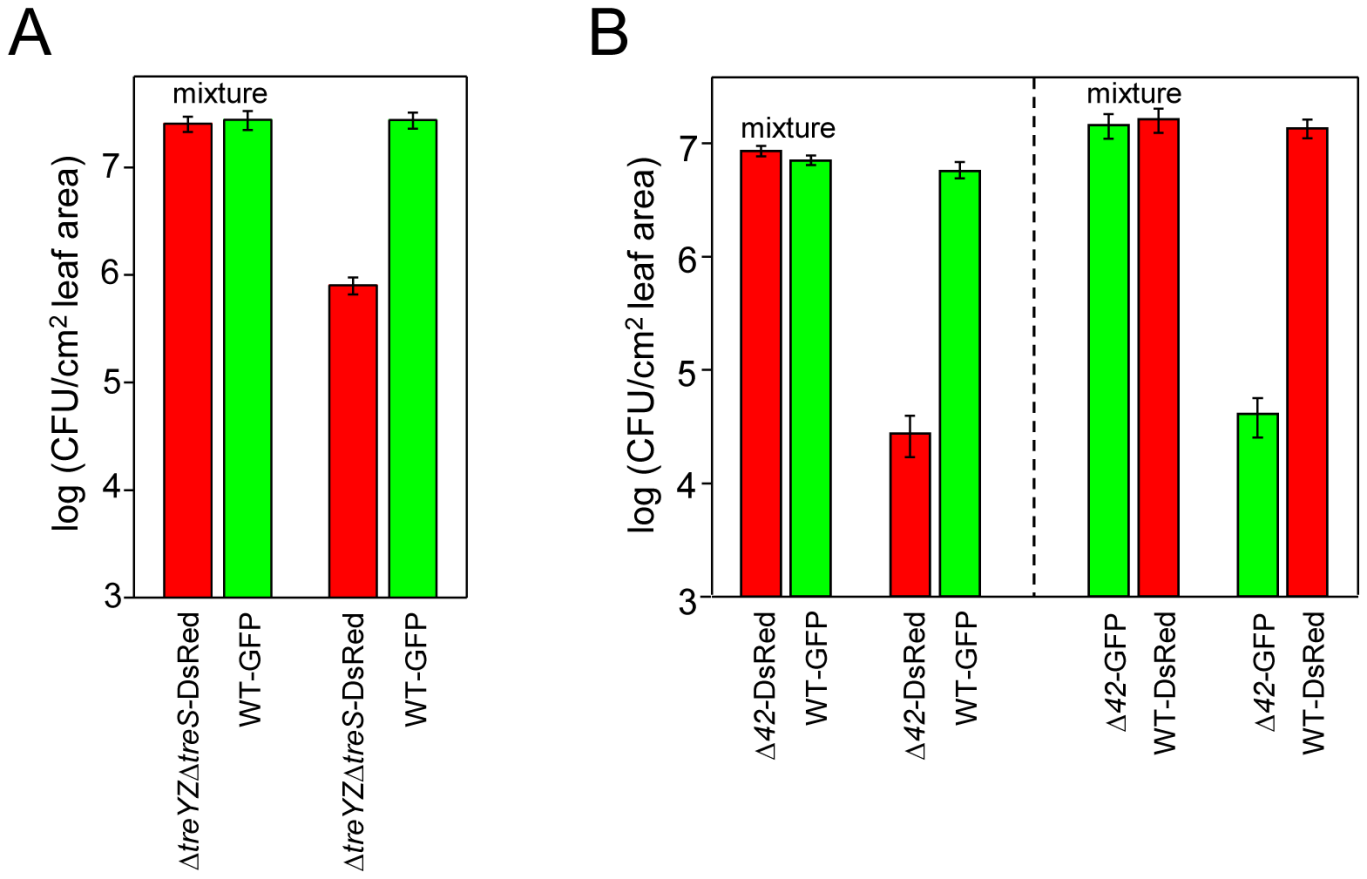
PA14 trehalose mutants are rescued in trans *in planta* by wild-type PA14. (A) Four-week-old Arabidopsis Col-0 plants were inoculated with a 1∶1 mixture of PA14 wild-type carrying pSMC2 (GFP) and the *ΔtreYZΔtreS* mutant carrying pAA100 (DsRed) at a total concentration of 3.5×10^2^ CFU/cm^2^ leaf area. As a control the strains were inoculated individually. (B) In the left panel, four-week-old Arabidopsis Col-0 plants were inoculated with a 1∶1 mixture of PA14 wild-type carrying pSMC2 (GFP) and the Δ*42* mutant carrying pAA100 (DsRed) at a total concentration of 6×10^2^ CFU/cm^2^ leaf area. As a control the strains were inoculated individually. In the right panel, PA14 wild-type carried pAA100 and the Δ*42* mutant carried pSMC2. Three days post infiltration, leaves were harvested and CFU determined by counting colonies using a Zeiss Stemi SV6 dissecting microscope fitted with a dual GFP/RFP filter. Data represent the mean of bacterial titers ± SE of six leaf disks excised from 6 leaves of 3 plants and are representative of three independent experiments.

The data in the previous section suggest that trehalose does not function intracellularly to protect PA14 from a variety of stresses during free-living growth. To provide evidence that trehalose functions extracellularly, we tested whether wild-type PA14 “complements” the growth defect of PA14 trehalose mutants *in planta*. Specifically, we co-inoculated Arabidopsis leaves with equal mixtures of wild-type PA14 and the Δ*treYZΔtreS* double trehalose mutant carrying plasmids that express GFP or DsRed, respectively (Figure 9A). Dramatically, co-inoculation of wild-type PA14 with Δ*treYZΔtreS* completely rescued the growth defect of Δ*treYZΔtreS* (Figure 9A), strongly suggesting that trehalose is most likely acting extracellularly and not internally within PA14 cells. Similar results were obtained when PA14 expressing GFP was mixed with the Δ*42* mutant expressing DsRed (Figure 9B, left panel). In this latter experiment, to make sure that the expression of red or green fluorescent protein does not affect bacterial strain viability, we also carried out an experiment in which the plasmids expressing fluorescent proteins were switched in wild-type PA14 and the Δ*42* mutant and obtained the same result (Figure 9B, right panel).

## Discussion

In this study, we report that synthesis of the disaccharide trehalose by the multi-host opportunistic pathogen *P. aeruginosa* is required for plant pathogenesis, but not for pathogenesis in at least three metazoan hosts, mice, *D. melanogaster* or *C. elegans*. Trehalose has been extensively characterized as a stress-response molecule that protects cells from osmotic, oxidative, and other environmental stresses. Surprisingly, however, our data suggest that trehalose does not function internally in *P. aeruginosa* to alleviate a variety of stresses that *P. aeruginosa* might encounter in its interaction with a plant host. Trehalose also does not function as a major osmoprotectant molecule for *P. aeruginosa*. Instead, because nitrate and ammonium ions, but not glucose, sucrose, or betaine, suppress the non-pathogenic phenotype of trehalose mutants *in planta*, we propose that trehalose may function to promote the acquisition of nitrogen-containing nutrients, thereby allowing *P. aeruginosa* to replicate in the intercellular spaces in a leaf. Moreover, because Arabidopsis cell wall mutants also suppress the non-pathogenic phenotype of the trehalose mutants, it is possible that the plant cell wall normally functions directly or indirectly as a barrier to block nutrient uptake by extracellular bacteria.

### Trehalose as a virulence factor for plant and animal pathogens

An important result from this work is that in contrast to many other bacteria and fungi, trehalose appears to have very little effect on protecting *P. aeruginosa* PA14 from a variety of diverse stresses, including osmotic, oxidative, pH, antibiotic, and temperature stress, and yet trehalose mutants are highly impaired in virulence in Arabidopsis. Instead of trehalose, our data show that *N*-acetylglutaminylglutamine amide (NAGGN) and glycine-betaine appear to be the primary stress response molecules in *P. aeruginosa*, in agreement with published data showing that osmotically stressed *P. aeruginosa* cultures accumulate NAGGN and glycine-betaine [27], [29]. Specifically, we found that a Δ*NAGGN* mutant was highly impaired in growth under osmotic stress and that exogenously added glycine-betaine, but not trehalose, protected Δ*42*, *ΔNAGGN*, and wild-type PA14 from osmotic stress (Figures 8A and S10A). Importantly, however, even though glycine-betaine is a potent stress protection molecule *in vitro*, it did not rescue the Δ*42* mutant *in vivo* (Figure 8B). Conversely, the Δ*NAGGN* or a *zwf* mutant, which are highly susceptible to osmotic or oxidative stress, respectively, were not impaired in plant infection (Table S1). These data suggest that trehalose does not play a role as a stress protection molecule in *P. aeruginosa* during plant infection.

Does trehalose function as a virulence factor for other bacterial phytopathogens in addition to *P. aeruginosa*? As shown in Table S2, the *treYZ* and *treS* trehalose biosynthetic operons are highly conserved among pseudomonads, including *P. syringae*, but it is not known whether trehalose functions as a virulence factor in these species. A recent study showed that deletion of *P. syringae* trehalose biosynthetic genes resulted in lowered fitness on the surface of plant leaves, but whether this was due to reduced virulence or increased susceptibility to hyperosmotic stress is not known [17].

What is the explanation for our observation that *P. aeruginosa* does not require trehalose for pathogenesis in at least three diverse metazoan hosts (mice, insects, and nematodes), and in fact may be a detriment for infection? In contrast to plants, mammals do not synthesize trehalose [30], and it is likely that trehalose, which is a stable and non-reactive molecule, has little effect on mammalian cells, at least at relatively modest concentrations. In the case of insects, trehalose is a major component of the hemolymph. Trehalose is also synthesized by *C. elegans*, where it accumulates during the formation of desiccation-resistant dauer larvae [31] and exogenous trehalose promotes *C. elegans* longevity [32]. Thus in the case of flies and worms, trehalose is beneficial and it appears unlikely that the additional levels of trehalose that are synthesized by *P. aeruginosa* would have a significant physiological affect. The apparent hypervirulence of the trehalose mutants in metazoan models of infection may simply be the result of increased fitness of the strain, which conserves energy by not synthesizing trehalose.

### Role of plant cell walls in PA14 infection

Because Arabidopsis cell wall mutants suppress the non-pathogenic phenotype of trehalose mutants, it seems likely that the virulence-enhancing role of trehalose is mediated through the plant cell wall. Can we attribute the lack of a particular plant cell wall polymer as playing a key role in the suppression of the non-pathogenic phenotype of the trehalose mutants? As shown in Figures 4 and S7, several Arabidopsis mutants that we tested either completely (*xxt1/xxt2* and *mur4-1*) or partially (*mur2-1*, *mur3-2*, and *mur10-2*) suppressed the phenotype of the trehalose mutants. A common feature of all of the cell wall mutants that we tested (Figures 4 and S6) is that they exhibit alterations in xyloglucan, the most abundant hemicellulose in the walls of dicotyledonous plants. The *xxt1/xxt2* mutant completely lacks xyloglucan [33], *mur2-1* and *mur3-2* display altered side chains in xyloglucan [34], [35], *mur4-1* has decreased levels of arabinose in xyloglucan [23], and *mur10-2* exhibits alterations in xyloglucan remodeling throughout the plant [36]. Interestingly, wild-type PA14 grew significantly less in *mur3-2* than in Col-0 plants, showing that *mur3-2* is more resistant to PA14 than wild-type plants. Thus, the fact that the trehalose mutants grew to the same extent in *mur3-2* as in Col-0 (Figures 4 and S6) suggests that *mur3-2* also partially suppresses its growth defect. These data suggest that xyloglucan may be a key component of the cell wall that affects the virulence of *P. aeruginosa*.

At the mechanistic level, it is not necessarily the case that the rigid plant cell wall is functioning, for example, simply as a physical barrier that blocks the ability of *P. aeruginosa* to extract nutrients from the cytoplasm of mesophyll cells. If the primary role of trehalose is to facilitate nutrient uptake, the source of the nutrients could be the apoplastic fluid or even components of the cell wall itself, such as specific cell-wall associated proteins.

Importantly, the enhanced susceptibility of the Arabidopsis cell wall mutants to the *P. aeruginosa* trehalose mutants is not simply a consequence of enhanced susceptibility to pathogens in general or the inability of the cell wall mutants to elicit an effective defense response. As shown in Figure 5, the cell wall mutants are not more susceptible to virulent or non-pathogenic *P. syringae* strains and appear to mount an effective innate immune response when challenged with the flagellin peptide flg22.

### How does trehalose promote *P. aeruginosa* virulence?

Specialized bacterial foliar phytopathogens primarily replicate in the intercellular spaces between mesophyll cells. It is poorly understood which plant-derived nutrients are critical for bacterial growth in this environment as well as the mechanisms utilized by pathogens to obtain nutrients from their hosts. The majority of these pathogens utilize type III effectors not only to suppress the host innate immune response [37], [38] but also to extract nutrients [10] from mesophyll cells. Interestingly, however, the *P. aeruginosa* type III secretion system is not necessary for pathogenesis in plants [6] and it seems unlikely that a broad host range pathogen such as *P. aeruginosa* would encode host-specific effectors that subvert the Arabidopsis sugar export system analogously to the *Xanthomonas* effectors that activate glucose efflux in mesophyll cells [10]. Indeed, it also seems highly unlikely that any particular *P. aeruginosa* strain has extensively co-evolved with any particular host [4], [9]. Instead, our finding that *P. aeruginosa* utilizes trehalose as a major virulence factor for plant pathogenesis is consistent with our studies with *P. aeruginosa* as a *C. elegans* pathogen, which have shown that the majority of virulence related factors required to infect nematodes correspond to genes that encode conserved global transcriptional regulators or “house-keeping” genes that encode enzymes involved in conserved metabolic processes [4], [9]. Trehalose biosynthesis is highly conserved. All pseudomonads (Table S2) and at least 30% of sequenced prokaryotic genomes encode presumptive trehalose biosynthetic enzymes (J. Urbach and F. Ausubel, unpublished data). It appears that *P. aeruginosa* has capitalized on what is mostly likely an ancient biosynthetic pathway to promote plant pathogenesis.

How does trehalose promote pathogenesis in an Arabidopsis leaf? Trehalose can serve as a carbon and energy source for growth of many bacteria and fungi including *P. aeruginosa* [25], [39]. However, we have shown that sucrose and glucose do not suppress the phenotype of *P. aeruginosa* trehalose mutants and co-inoculation of a double Δ*42treA::MAR2xT7* mutant (which cannot metabolize trehalose) with trehalose rescues the non-pathogenic phenotype similarly as co-inoculation of the Δ*42* mutant with trehalose (Figure S6). These data suggest that either the level of carbon is not limiting or that trehalose is not involved in carbon acquisition. In addition, experiments designed to determine whether trehalose promotes activity of CWDEs failed to provide evidence that trehalose plays a significant role in cell wall degradation, with the caveats, however, that the experiments we carried out were done *in vitro* with commercial CWDEs and that in our particular assay a relatively low level of trehalose – enhanced hydrolysis would have not been detected.

Does trehalose function as a general toxin to disrupt host cellular processes? A number of studies have shown that exogenously applied trehalose can have a major negative impact on seedling growth and development [40]–[42]. On the other hand, the concentrations of trehalose used in these seedling experiments (from 30 mM to 100 mM) were significantly higher than the levels that would be expected to be encountered under natural conditions. By way of contrast, in our experiments we used mature four-week old plants and substantially lower concentrations of trehalose (most often 1 mM). In mature plants, trehalose did not have a toxic effect as evidenced by the lack of any visible symptoms following trehalose (1 mM) infiltration (data not shown). Importantly, in our experiments, trehalose concentrations as low as 0.74 mM largely suppressed the non-pathogenic phenotype of the Δ*42* mutant and 0.074 mM had a significant effect (Figure 2C). Taken together, our data indicate that trehalose does not have a toxic effect on mature plants in the *P. aeruginosa* - plant infection model.

Does trehalose upregulate PA14 virulence genes expression? If so, it would have to specifically upregulate genes required for plant pathogenesis because as shown in Figures 3 and S5, PA14 trehalose mutants are not less virulent in nematodes, flies, or mice. However, we do not favor this explanation. As shown in Figures 6, S8, and S9, nitrate, ammonium, maltose and maltotriose functioned similarly to trehalose in suppressing the inability of the PA14 trehalose mutants to grow *in planta*. It seemly highly unlikely that all three sugars as well as nitrate and ammonium would function similarly to each other as signaling molecules.

Since the non-pathogenic phenotype of *P. aeruginosa* trehalose mutants can also be suppressed by ammonium nitrate, we propose that trehalose promotes the acquisition of nitrogenous compounds and that nitrogen is limiting in the intercellular environment. The intercellular spaces in leaves are mostly filled with air [43] and very little is known about the mechanisms that plant pathogens utilize to obtain nutrients in this dry environment. Nitrogen limitation during *P. aeruginosa* infection in plants has been reported previously [44], [45]. One way that trehalose could promote nitrogen acquisition is by modulating host nitrogen metabolism, thereby diverting nitrogen-containing compounds to invading *P. aeruginosa* cells. Several *in planta* studies have shown that trehalose-6-phosphate (T6P) plays a key role in the regulation of carbon and nitrogen metabolism [41], [46], [47] and is associated with altered cell wall structure and starch accumulation [48]–[50]. In our study, preliminary transcriptional profiling analysis has shown that infiltration of trehalose into Arabidopsis leaves at a concentration that is effective in rescuing the trehalose mutants (1 mM) has only a very modest effect on Arabidopsis gene expression (S. Djonovic and F. Ausubel, unpublished data). In addition, we showed that trehalose or ammonium nitrate does not modulate plant defense responses, since infiltration of ammonium nitrate or trehalose into Arabidopsis leaves did not make them more susceptible or resistant to virulent or non-pathogenic *P. syringae* strains or interfere with their ability to mount an effective innate immune response when challenged with the flagellin peptide flg22 (Figure 7). Finally, another way that *P. aeruginosa* could use trehalose to promote nitrogen acquisition is by generating a high local concentration of trehalose to create an osmotic gradient that causes an efflux of nitrogen containing nutrients from neighboring plant cells, perhaps in conjunction with *P. aeruginosa*-encoded pore forming toxins. The data in Figure 9, which shows that trehalose functions externally to *P. aeruginosa*, is consistent with these proposed models.

### Conclusions

We have found that *P. aeruginosa*-synthesized trehalose plays a key role as a virulence factor during infection of plant leaves. Although the mechanistic details remain to be elucidated, our data suggest that a role of trehalose during the infectious process involves the procurement of nitrogen-containing molecules. In contrast to specialized plant pathogens that utilize highly evolved Type III virulence effectors to promote virulence, the multi-host opportunistic pathogen *P. aeruginosa*, which is not likely to have co-evolved with particular plant hosts, appears to have repurposed a highly conserved anabolic pathway (trehalose biosynthesis) as a potent virulence factor.

## Materials and Methods

### Ethics statement

Experiments with mice were carried out in strict accordance with the recommendations in the Guide for the Care and Use of Laboratory Animals of the National Institutes of Health. The animal protocol was approved by the Harvard Medical Area Institutional Animal Care and Use Committee (Permit Number: 404). All efforts were made to minimize suffering.

### Bacterial strains and media

*P. aeruginosa* strain UCBPP-PA14 [1], *P. syringae* pv. *tomato* strain DC3000 [51], and *P. syringae* pv. *phaseolicola* strain 3121 [52] have been described. A nonpolar *hrcC* mutant of *P. syringae* strain DC3000 (CUCPB5112) was obtained from A. Collmer and B. Kvitklo, Cornell University. *Escherichia coli* strain SM10 λpir was used for triparental mating [53]. Strains were routinely maintained at 37°C on Luria-Bertani (LB) agar plates or cultured in LB broth supplemented with appropriate antibiotics as needed. The concentrations of antibiotics were: ampicillin or carbenicillin, 50 µg/ml for *E. coli* or 300 µg/ml for *P. aeruginosa*; and rifampicin 100 µg/ml. Minimal medium (M63) or modified minimal A medium (MinA) that contained glucose (0.3%) [39] were also used for the growth of *P. aeruginosa*.

### Generation of in-frame PA14 deletion mutants

The Δ*PA14_36375-36830* deletion mutant (Δ*42*) was constructed using a 2.25 kb sequence containing regions immediately flanking the deleted region that was generated by a standard 3-step PCR protocol using FastStart Taq DNA Polymerase (Roche, Germany) and cloned into the *Kpn*I and *Bam*HI sites of pEX18Ap [54] creating plasmid pEX18PA14_36375-36830Δ1. The resulting plasmid was used to introduce the deleted PA14_36375-36830 region into the wild-type PA14 genome by homologous recombination [53]. Similar strategies were used to construct other deletion mutants. For Δ*PA14_36375-36560, ΔPA14_36570-36630, ΔPA14_36570-36700, ΔPA14_36710-36740, ΔPA14_36710-36830*, and *ΔPA14_19350-19370*, 12.64-, 10.58-, 16.28-, 7.50-, 12.81-, and 4.78 kb wild-type sequences were deleted by recombination using 1.30, 1.27, 1.06, 1.28, 1.30, and 1.26 kb fragments, respectively, containing the relevant flanking sequences. A double mutant lacking both trehalose operons was constructed by recombining the deleted *treS* operon in pEX18PA14_36710-36830Δ1 into the *ΔPA14_36570-36700* (*treYZ*) mutant background. A double Δ*42treA* mutant was constructed by recombining the Δ*42* deletion in pEX18PA14_36375-830Δ1 into a *treA*::*MR2xT7* transposon insertion mutant [16]. We confirmed that *treA*::*MR2xT7* mutant could not grow when provided trehalose as sole carbon source in diluted LB and that *treA::MR2x7* could not hydrolyze trehalose to glucose using the Somogyi-Nelson assay [21], [22]. All deletion mutants were confirmed by PCR analysis and sequencing.

### Generation of fluorescently labeled bacterial strains

PA14 wild-type and the Δ*42* mutant were transformed with pSMC2 carrying green fluorescent protein (GFP) [55]. To construct strains expressing red fluorescent protein (RFP), a variant of DsRed2, DsRed.T3(DNT), from *Vibrio fischeri* [56] was transferred (on a 719 bp *Sph*I – *Xba*I fragment from pVSV208) into the *Sph*I – *Xba*I sites of pUCP19 [57] generating pUCP19/DsRed.T3(DNT), which was designated pAA100. pAA100 was transformed into PA14 wild-type and Δ*42* by electroporation.

### Motility and growth assays

Twitching and swimming motility assays were performed as previously described [58]. To compare growth rates of wild-type and mutants, the cultures were grown at 37°C overnight in LB, centrifuged, washed and resuspended into minimal medium (M63). Bacterial growth was monitored *in vitro* by plating and counting CFU/ml at 3-, 6- and 9- hour time points. Growth rate (h^−1^) was calculated using the equation for exponential growth: *μ* = (ln*N*_1_−ln*N*_0_)/(*t*_1_−*t*_0_), where *N*_0_ and *N*_1_ equal bacterial abundance (CFU/ml) at the beginning (*t*_0_) and end (*t*_1_) of the exponential growth phase. Each experiment was repeated at least twice with similar results.

### Plant material and growth of plants

Arabidopsis ecotypes Columbia (Col-0) was obtained from the Arabidopsis Biological Resource Center (Columbus, OH). Plants were grown on 30-mm Jiffy-7 peat pellets (Jiffy Products, Shippagan, New Brunswick, Canada) in a Conviron E7/2 chamber (Winnipeg, Manitoba, Canada) set at a 23°C/20°C day/night regime with a 12-h photoperiod at a light intensity of 100 µE m^−2^ s^−1^ and 60% relative humidity. Arabidopsis cell wall mutants were obtained from the Arabidopsis Biological Resource Center: *mur2-1* (AT2G03220; CS8565), *mur3-2* (AT2G20370, CS8567), *mur4-1* (at1g30620, CS8568), *mur10-2* (at5g17420, CS8578), and an *xxt1/xxt2* double T-DNA insertion line (at3g6272; SALK_119658C/at4g02500; SALK_1013080) as previously published [33].

### Arabidopsis pathogenicity assays

Plant infection assays were carried out as previously described [1] with some modifications. *P. aeruginosa* strains were grown in LB medium overnight, subcultured and grown to an OD_600_ of 2.5. Cells were centrifuged, washed and resuspended in 10 mM MgSO_4_. Leaves of four-week old plants were inoculated with a 1×10^5^ CFU/ml suspension of PA14 wild-type or various PA14 mutants, which corresponds to 1×10^3^ CFU/cm^2^ leaf area. Infected plants were incubated in a growth chamber at 28°C with a 12-h photoperiod at a light intensity of 60 µE m^−2^s^−1^ and 90% relative humidity. Six to eight leaves were harvested from three to four plants for CFU determination. Each experiment was repeated at least two to four times with similar results. Co-inoculation of bacteria with betaine, trehalose or sucrose (Sigma, St. Louis, MO) was performed as described above. Before inoculation of leaves, betaine, trehalose, glucose, or sucrose was added to bacterial suspensions at the indicated concentrations, or various phosphate, sulfate, nitrate, or ammonium salts were added at 1 mM. Trehalose was initially added at 2.5 mg/ml, but after we carried out dose response curves and found that 0.25 mg/ml (0.74 mM) was an effective concentration, subsequent experiments were carried out using 1 mM trehalose.

Infection assays with *P. syringae* strains were performed the same way as with *P. aeruginosa* with a few exceptions. The temperature in the growth chamber was 22°C and bacterial strains were cultured in King's B medium (protease peptone, 10 mg/ml; glycerol, 15 mg/ml; K_2_HPO_4_, 1.5 mg/ml; MgSO_4_, 5 mM, pH 7.0) until late logarithmic phase. Elicitation assays were performed by infiltration of leaves with 1 µM flg22, 1 mM trehalose, 1 mM glucose or 1 mM ammonium nitrate (or in combinations) 24 hours prior to bacterial inoculation.

### Metazoan pathogenicity assays

*C. elegans* slow killing assays were performed as previously described [3]. Briefly, PA14, Δ*42*, or Δ*treYZ*Δ*treS* mutants were grown overnight in LB and 10 µl of each liquid culture was spread onto 3 SK plates (modified NGM medium; [3]). The plates were incubated at 37°C for 24 hours and then at 25°C for 20–24 hours. 35–45 *fer-15;fem-1* sterile L4 nematodes were picked to the SK plates seeded with PA14, Δ*42*, or Δ*treYZ*Δ*treS* and the plates were incubated at 25°C. Live and dead animals were counted daily for approximately 8 days. A worm was scored dead when it no longer responded to touch.

Infection survival assays in *D. melanogaster* were conducted with *D. melanogaster* strains w[118] (Bloomington stock #6326) or Oregon R, which were grown under non-crowded conditions on standard cornmeal-molasses medium. Fly husbandry and infections were carried out at 25°C, 70% humidity, 12 hours light cycle. For infections assays, *P. aeruginosa* was grown aerated at 37°C in LB medium containing 50 µg/ml rifampicin, and subcultured to an OD_600_ = 2.3–2.5. The bacterial culture was diluted to a final concentration 80% LB, 4% sucrose, 50 µg/ml rifampicin and 3×10^8^ CFU/ml and 7 ml of infection mixture was pipetted onto sterilized cotton balls at the bottom of clean, empty fly vials. 25 male flies, 4 days old, were added and their survival monitored several times a day.

Mouse experiments complied with institutional and federal guidelines regarding the use of animals in research. For the acute pneumonia model, a modified version of a previously described method of intranasal inoculation of anesthetized mice was utilized [19]. Briefly, 6- to 8-week-old female C3H/HeN mice (Harlan) were sedated with ketamine and xylazine and then 10 µl of a bacterial suspension was applied to each nostril. Bacterial suspensions were prepared in PBS (OD_600_ = 0.5) after overnight growth of frozen stock on TSA. Doses were determined by serial dilution and plating on MacConkey agar (1.5×10^7^ CFU/20 µl for PA14, 1.4×10^7^ CFU/20 µl for Δ*42*). After 18 hr, mice were euthanized with carbon dioxide and then lungs and spleens were removed, weighed, and homogenized in 1 ml of 1% proteose peptone in water. Viable counts were determined by serial dilution and plating.

For the chronic oropharyngeal colonization model in transgenic CF mice, we utilized mouse strain *Cftr* ^tm1Unc^-TgN^(FABP-CFTR)^ (denoted FABP-CFTR), which has a stop codon in the murine *cftr* gene (S489X) but also expresses human CFTR in the gut epithelium due to transgenic introduction of human *Cftr* under the control of the fatty acid binding protein (FABP) promoter [59]. These FABP-CFTR mice have been bred into the FVB/N genetic background (breeding pairs were initially provided by Dr. J. Whitsett, University of Cincinnati). These FABP-CFTR mice are susceptible to chronic oropharyngeal colonization with *P. aeruginosa* after exposure in the drinking water [20]. To establish colonization, age- and gender-matched mice were given oral levofloxacin in their drinking water for 5 days, followed by gentamicin for 2 days, followed by bacteria (either PA14 or the Δ*42* mutant) suspended in water at 10^7^ CFU/ml. Bacterial levels in the drinking water were unchanged at the end of 7-day exposure. Throat cultures were then taken every 1–2 weeks using a swab inserted into the oropharynx of mice anesthetized with isofluorane. The swab was placed in 1 ml tryptic soy broth and incubated at 37°C for 3 hours. Next, 1 ml of nitrofurantoin (2 mg/ml) was added to suppress the growth of any contaminating *Enterobacter spp*., which can interfere with detection of *P. aeruginosa*. The culture was incubated overnight at 37°C and then subcultured overnight on cetrimide agar. All mice in both groups (n = 9 for the Δ*42* mutant, n = 8 for PA14 WT) had positive throat cultures after colonization. Mice were then followed for survival.

### Bioinformatic analysis of PA14_36375-36830 genes

To assign putative functions to genes within the block of PA14_36375 through PA14_36830, each protein in the 42 kb cluster was used as a query in a BLAST or PSI-BLAST homology search. In the process of assigning putative functions, several types of information were taken into account: homologous proteins with experimentally assigned function; homologous proteins with computationally predicted function; matches to HMMs from conserved domain databases; and the genomic/operon context of close homologs.

The protein and nucleotide sequences of prokaryotic genomes were obtained from NCBI (ftp://ftp.ncbi.nih.gov/genomes/Bacteria/). Additionally, two *P. aeruginosa* genomes (*P. aeruginosa* 2192; *P. aeruginosa* C3719) were obtained from the Broad Institute of Harvard and MIT (http://www.broadinstitute.org/annotation/genome/pseudomonas_group/MultiHome.html).

To identify orthologs to PA14_36375-36830 genes, two criteria were used. First, putative ortholog pairs were required to be reciprocal best hits, with an e-value less than or equal to 0.0001 for best hits of the PA14 proteins against compared protein sets, and an e-value less than or equal to 0.001 for reciprocal best hits against the PA14 protein set. Secondly, the putative orthologs were required to align for at least 80 percent of their lengths and have less than a 20% difference in protein sequence lengths, thereby conserving overall domain structure. Of these constraints, the e-value and sequence length constraints are very permissive, whereas the requirement for alignment length is stringent.

### Trehalose quantification assay

*P. aeruginosa* strains were grown at 37°C in MinA medium with 0.5 M NaCl to an early stationary phase. Trehalose was extracted from a 19 ml culture by pelleting the cells, resuspending in 0.5 ml water, and heating at 95°C for 20 min [60]. The concentration of trehalose in the supernatants was determined using an enzymatic assay by converting trehalose to glucose with trehalase and then measuring the glucose using a trehalose assay kit (Megazyme International Ireland Limited). The pre-existing glucose in each sample was determined in a control reaction without trehalase and subtracted from the total glucose. The experiment was repeated at least twice with similar results.

### Stress response, resistance and biofilm assays

*Osmotic stress sensitivity*: *P. aeruginosa* was grown at 37°C in MinA containing 17 mM glucose [39], washed and subcultured into MinA containing 0.5 M NaCl. Bacterial growth was monitored by plating CFU. *Persistence assay:* This assay was performed as previously described [61]. Briefly, persisters were determined by exposure of stationary cultures to antibiotics at concentrations exceeding the corresponding bacterial minimal inhibitory concentrations (MICs). The antibiotics were used at the following concentrations: 6 µg/ml tobramycin, 2 µg/ml ciprofloxacin and 3 mg/ml carbenicillin. *Oxidative stress resistance:* Hydrogen peroxide was added directly to an overnight culture grown for 14 h in LB medium and the cells were incubated for 8 h at 37°C. The following concentrations of hydrogen peroxide were used: 1 M (non-lethal), 2 M (sub-lethal), and 3 M (lethal dose). To test sensitivity to paraquat (Sigma, 856177), overnight MinA cultures were diluted 100 fold in MinA containing different amounts of paraquat (0.1, 1 and 10 mM) and cultured for three days. *pH stress:* Cultures were grown to stationary phase in LB medium that had been titrated with HCl to pH 4, 5, 6, or 7. *Thermotolerance:* Small volumes of stationary phase cells were heated in Eppendorf tubes in a heating block at different temperatures and incubation times, then rapidly diluted and plated. *Biofilm formation:* Biofilm attachment assays were performed using wild-type PA14, Δ*42* and *ΔPA14_19350-19370* (Δ*NAGGN*) cultures grown in 96-well polyvinylchloride (PVC) plates as described previously [62]. Overnight cultures were diluted 1/100 in MinA medium or MinA medium supplemented with 0.5 M or 0.75 M NaCl. Aliquots of 100 µL were dispensed into the wells of PVC microtiter plates and incubated at 37°C. Attachment was detected by staining with 1% crystal violet dissolved in water. Dye not associated with bacteria was removed by rinsing with water. Bacteria-associated dye was solubilized using 95% ethanol and absorbance was determined at 550 nm. Each experiment was repeated at least twice with similar results.

### Statistical analysis

Statistical analyses in animal experiments were performed using GraphPad Prism 5 software (La Jolla, CA) and a log rank (Mantel-Cox) test to assess the significance of differential survival, and a Mann-Whitney U non-parametric test for significance of CFU data, which were not normally distributed. Statistics in all other experiments was performed using analysis of variance (ANOVA) and a Fisher's PLSD test (Statview v. 5.0.1, SAS Institute, Cary, NC).

## Supporting Information

Figure S1 Expression of PA14_36500 encoding a putative cellulase/peptidase in infected Arabidopsis leaves. Semiquantitative RT-PCR was carried out as described in Materials and Methods. (A) PA14_36500 transcript levels on various days post infiltration (dpi) with PA14 wild-type. (B) PA14_36500 expression in wild-type PA14 and a PA14 Δ*mvfR* mutant 2 days post-infiltration. *P. aeruginosa* PA14 ribosomal protein L21 (*rplU*) was used as a control for equal amounts of cDNA. The experiment was repeated at least two times with similar results.
(TIF)

Figure S2 The 42 kb cluster encodes two independent pathways for trehalose biosynthesis. In the top pathway, gene product 36605 (a putative maltooligosyltrehalose synthase) alters the regiochemistry of the terminal sugar linkage from alpha-1,4 to alpha-1,1; the terminal disaccharide is subsequently cleaved by gene product 36580 (a putative maltooligosyltrehalose trehalohydrolase), releasing trehalose. In the bottom pathway, gene product 36740 (a putative alpha-amylase) cleaves the terminal disaccharide of the alpha-1,4-glucan, releasing maltose. The alpha-1,4 linkage of the maltose disaccharide is then isomerized to alpha-1,1 by gene product 36730 (a putative trehalose synthase), yielding trehalose.
(TIF)

Figure S3 Pyocyanin production by Δ*42* and PA14 wild-type. Bacterial strains were streaked onto *Pseudomonas* agar P to assess pyocyanin production (see Materials and Methods) and incubated 20 h at 37°C. Sectors: Δ*42*-1 and Δ*42*-2 (two independent Δ*42* deletion constructs); *phzM* (*phzM::MAR2xT7*, negative control: a pyocyanin-defective mutant); WT (PA14 wild-type). Characteristic blue-green color indicates that the strain is proficient in pyocyanin production.
(TIF)

Figure S4 Growth of *P. aeruginosa* Δ*42*, three sub-region in-frame deletion mutants, and PA14 wild-type in Arabidopsis Col-0 leaves. Plants were inoculated and incubated as described in Materials and Methods. The leaves were harvested 3 days post-inoculation. Data represent the mean of bacterial titers ± SE of six leaf disks excised from 6 leaves of 3 plants. Different letters above bars denote statistically significant differences (P<0.05, Fisher's PLSD test). See Figure 1 for a description of the mutants.
(TIF)

Figure S5 The Δ42 mutant is more virulent than wild-type PA14 in nematodes, insects, and mice. (A) *C. elegans* are more sensitive to killing by the Δ*42* mutant than PA14 wild-type (P<0.004). Mutant *fer15;fem1 C. elegans* animals were exposed to *P. aeruginosa* strains and survival was determined as described in Materials and Methods. Data at each time point correspond to the average of three plates per strain, each with approximately 40 animals per plate, and are representative of two independent experiments. (B) *D. melanogaster* infected with Δ*42* die faster than flies infected with PA14 wild-type (P<0.03). *D. melanogaster* strain Oregon R was infected with *P. aeruginosa* and approximately 25 flies per vial were scored several times a day for survival throughout the time course of infection. Data are representative of four independent experiments carried out with two different *D. melanogaster* lines (Oregon R and w[118]). (C) The Δ*42* mutant is more virulent in a murine acute lung infection model. See Materials and Methods for details of infection protocol. CFU/gram of lung tissue of mice infected with Δ*42* mutant is 3.8-fold higher than with wild-type PA14 18 hours post intranasal infection (P<0.01, Mann-Whitney U test). Data are representative of two independent experiments. (D) FABP-CFTR transgenic mice are more susceptible to killing by Δ*42* mutant than by PA14 wild-type after oropharyngeal colonization (P<0.04, log rank test). All mice in both groups (n = 9 for Δ42; n = 8 for PA14 WT) had positive throat cultures for the duration of the experiment after initial colonization by exposure to bacteria in drinking water for one week.
(TIF)

Figure S6 The *in planta* growth defect of the Δ*42* mutant is suppressed by Arabidopsis cell wall mutants. Growth of PA14 wild-type or Δ*42* 3 days post infiltration in Arabidopsis cell wall mutants *mur2-1*, *mur3-2*, *mur4-1*, *mur10-2* and *xxt1/xxt2*. Data represent the mean of bacterial titers ± SE of six leaf disks excised from 6 leaves of 3 plants. Letters above bars denote statistically significant differences (P<0.05, Fisher's PLSD test). The experiments were repeated at least two times.
(TIF)

Figure S7 The growth of the Δ*42* mutant *in planta* is not limited by lack of a carbon source. (A) The growth of the Δ*42* mutant *in planta* is not suppressed by 1.25 mg/ml glucose or 2.5 mg/ml sucrose. (B) Suppression of the growth defect of Δ*42treA::MAR2xT7* with trehalose in Arabidopsis leaves. Plants were inoculated and incubated as described in Materials and Methods and leaves were harvested 3 days post-inoculation. In (A) and (B), data represent the mean of bacterial titers ± SE of six leaf disks excised from 6 leaves of 3 plants. Different letters above bars denote statistically significant differences (P<0.05, Fisher's PLSD test). The experiments were repeated at least two times.
(TIF)

Figure S8 The *in planta* growth defect of the Δ*42* mutant is suppressed by ammonium or nitrate ions. Leaves of four-week-old Arabidopsis Col-0 plants were infiltrated with PA14 wild-type or Δ*42* as described in Materials and Methods except that the infiltration solution contained various phosphate, sulfate, nitrate, or ammonium salts at 1 mM. Suppression of the growth defect of Δ*42* with 2.5 mg/ml trehalose (Tre) and 1.25 mg/ml glucose (Glc) were tested as positive and negative controls, respectively. Data represent the mean of bacterial titers ± SE of six leaf disks excised from 6 leaves of 3 plants. Letters above bars denote statistically significant differences (P<0.05, Fisher's PLSD test). The experiments were repeated at least two times.
(TIF)

Figure S9 Suppression of attenuation of Δ*42* with exogenous maltose and maltotriose. Col-0 plants were infiltrated with PA14 wild-type or Δ*42* co-inoculated with maltose (M) or malotriose (MT) at the indicated concentrations. Leaves were harvested 3 days post-infiltration. Data represent the mean of bacterial titers ± SE of six leaf disks excised from 6 leaves of 3 plants. Letters above bars denote statistically significant differences (P<0.05, Fisher's PLSD test). The experiment was repeated at least two times.
(TIF)

Figure S10 Response of Δ*42* to various stress conditions. (A) Rescue of Δ*42, ΔNAGGN* and PA14 wild-type with betaine but not trehalose under osmotic stress *in vitro*. Cells were grown at 37°C in MinA medium with 0.5 M NaCl (squares), or with 0.5 M NaCl+1 mM betaine (Bet) (circles) or 1 mM trehalose (Tre) (triangles). Data represent the mean ± SE of 3 replicates. (B) Thermotolerance. Survival of stationary phase bacteria after a 30 minute exposure to 53°C. Temperatures below 53°C were non-lethal and above 56°C were 100% lethal. (C) Biofilm attachment under osmotic stress. Overnight cultures were diluted 1/100 in MinA medium supplemented with 0.5 or 0.75 M NaCl. Attachment assays were performed as described in Materials and Methods. (D) Growth under pH stress. Cultures were grown to stationary phase in LB medium adjusted to pH 4, 5, 6, or 7. (E) Persistence assay. Persisters were determined by exposure of stationary cultures (inoculum, time point zero on x axis) to 6 µg/ml tobramycin, 2 µg/ml ciprofloxacin or 3 mg/ml carbenicillin. The assay was performed as described in Materials and Methods. Based on analysis of variance (ANOVA) and Fisher's PLSD test (P<0.05), there was no significant differences between Δ*42* and PA14 wild-type in any of the assays (A–E).
(TIF)

Table S1 Growth of *P. aeruginosa* mutants in Arabidopsis Col-0 leaves. Plants were inoculated and incubated as described in Materials and Methods and leaves were harvested 3 days post-inoculation. Data represent the mean of bacterial titers ± SE of six leaf disks excised from 6 leaves of 3 plants. Different superscript letters denote statistically significant differences (P<0.05, Fisher's PLSD test).
(DOC)

Table S2 Predicted functions and orthologs of PA14_36375-36830 genes across sequenced *Pseudomonas* genomes. Putative functions of PA14_36375-36830 genes were assigned as described in Materials and Methods. Genes of the reference taxon PA14 are indicated in the left column (PA14 loci), and different pseudomonad genomes are indicated on the top row. The presence of an ortholog is indicated by a checkmark. Boxes of identical hue indicate that the genes are contiguous in a particular genome, with the lighter shades on the top strand, and darker shades on the bottom strand. Checks in white boxes indicate an ortholog that is not contiguous with other PA14_36375-36830 block orthologs.
(PDF)

Table S3 Growth, biofilm formation and motility of Δ*42* and PA14 wild-type. Growth rate (h^−1^) of *Pseudomonas* strains in minimal media (M63) was calculated by the equation for exponential growth (see Materials and Methods). Biofilm formation was measured as attachment to polyvinylchloride plates in absorbance units (OD550; see Materials and Methods). Swimming and twitching motility are represented as a radius of a halo in cm (see Materials and Methods). Two independent Δ*42* deletion constructs were tested. Data represent the mean ± SE. Based on analysis of variance (ANOVA) and Fisher's PLSD test (P<0.05), there was no significant differences between Δ*42* mutant and PA14 wild-type in any of the assays.
(DOC)
