# Supplementary figures and images for: Trehalose Biosynthesis Promotes Pseudomonas aeruginosa Pathogenicity in Plants
Source: PLoS Pathog. 2013 Mar 7;9(3):e1003217. doi: 10.1371/journal.ppat.1003217 (PMC3591346; doi:10.1371/journal.ppat.1003217)

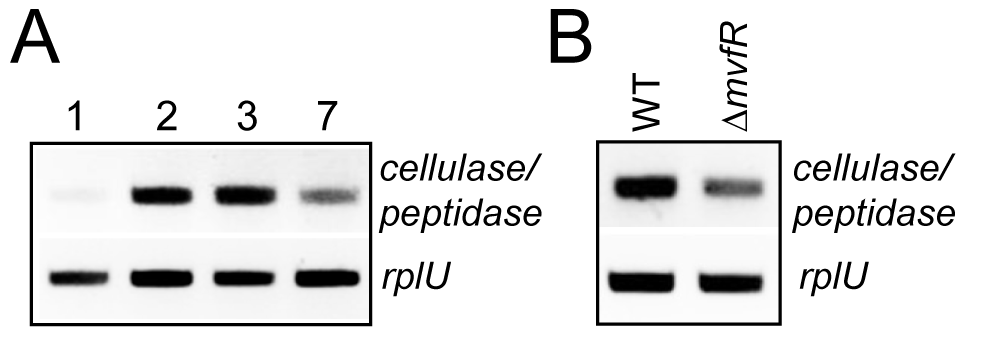

Supplement: Figure S1 — Expression of PA14_36500 encoding a putative cellulase/peptidase in infected Arabidopsis leaves. Semiquantitative RT-PCR was carried out as described in Materials and Methods. (A) PA14_36500 transcript levels on various days post infiltration (dpi) with PA14 wild-type. (B) PA14_36500 expression in wild-type PA14 and a PA14 ΔmvfR mutant 2 days post-infiltration. P. aeruginosa PA14 ribosomal protein L21 (rplU) was used as a control for equal amounts of cDNA. The experiment was repeated at least two times with similar results. (TIF) [file ppat.1003217.s001.tif]

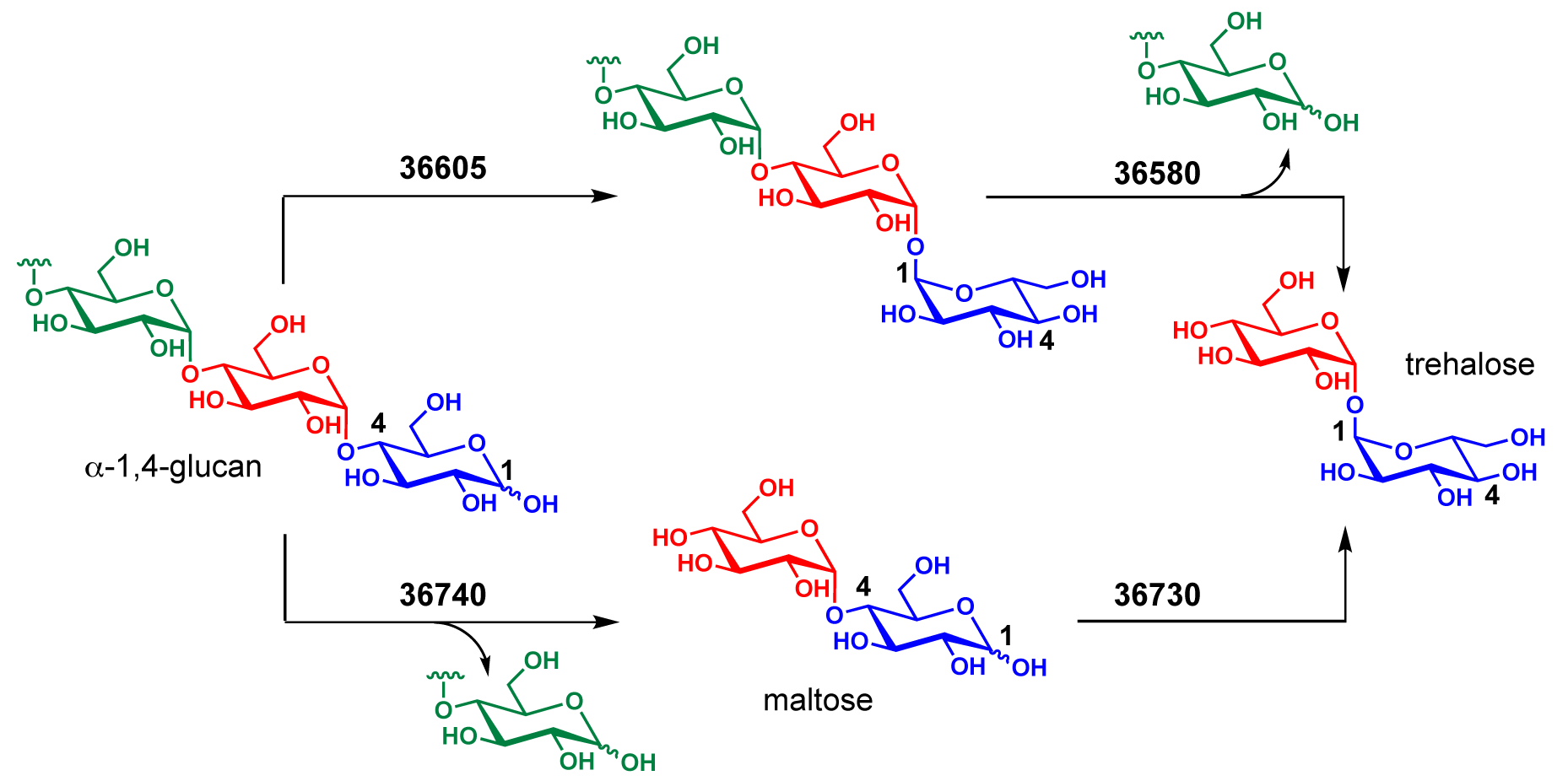

Supplement: Figure S2 — The 42 kb cluster encodes two independent pathways for trehalose biosynthesis. In the top pathway, gene product 36605 (a putative maltooligosyltrehalose synthase) alters the regiochemistry of the terminal sugar linkage from alpha-1,4 to alpha-1,1; the terminal disaccharide is subsequently cleaved by gene product 36580 (a putative maltooligosyltrehalose trehalohydrolase), releasing trehalose. In the bottom pathway, gene product 36740 (a putative alpha-amylase) cleaves the terminal disaccharide of the alpha-1,4-glucan, releasing maltose. The alpha-1,4 linkage of the maltose disaccharide is then isomerized to alpha-1,1 by gene product 36730 (a putative trehalose synthase), yielding trehalose. (TIF) [file ppat.1003217.s002.tif]

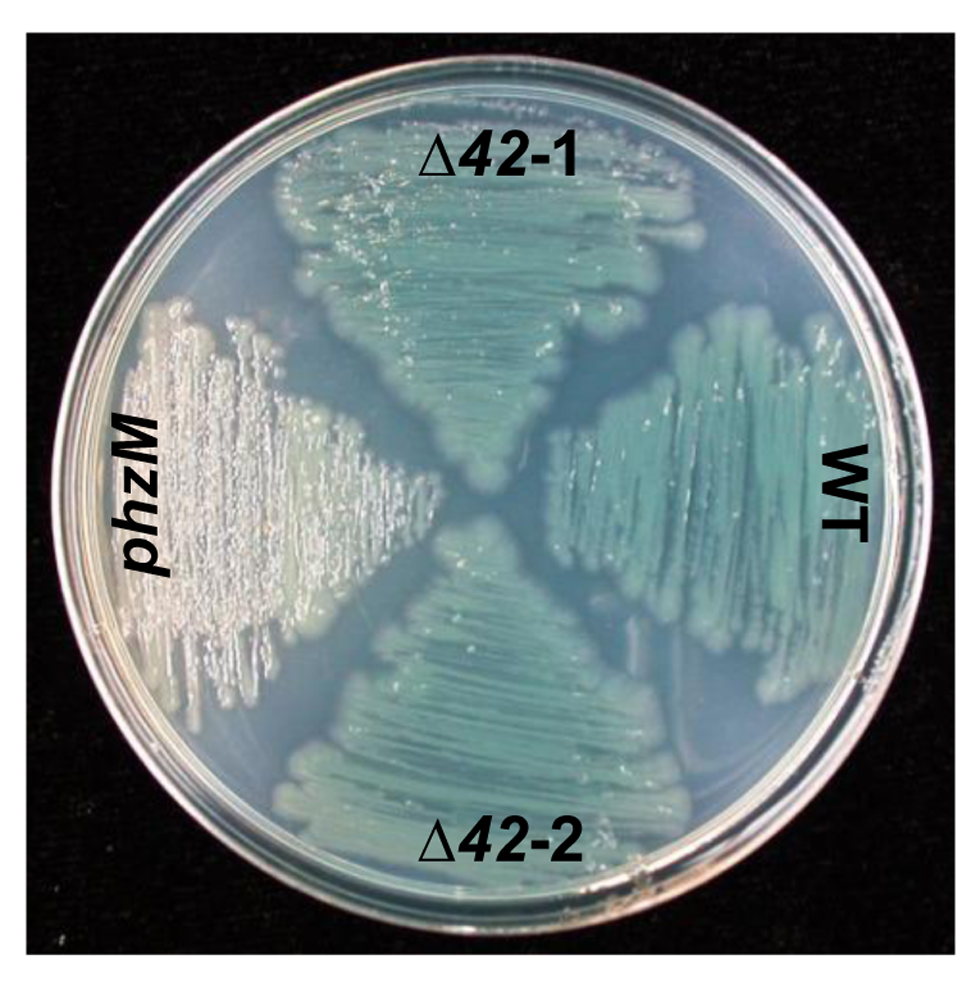

Supplement: Figure S3 — Pyocyanin production by Δ42 and PA14 wild-type. Bacterial strains were streaked onto Pseudomonas agar P to assess pyocyanin production (see Materials and Methods) and incubated 20 h at 37°C. Sectors: Δ42-1 and Δ42-2 (two independent Δ42 deletion constructs); phzM (phzM::MAR2xT7, negative control: a pyocyanin-defective mutant); WT (PA14 wild-type). Characteristic blue-green color indicates that the strain is proficient in pyocyanin production. (TIF) [file ppat.1003217.s003.tif]

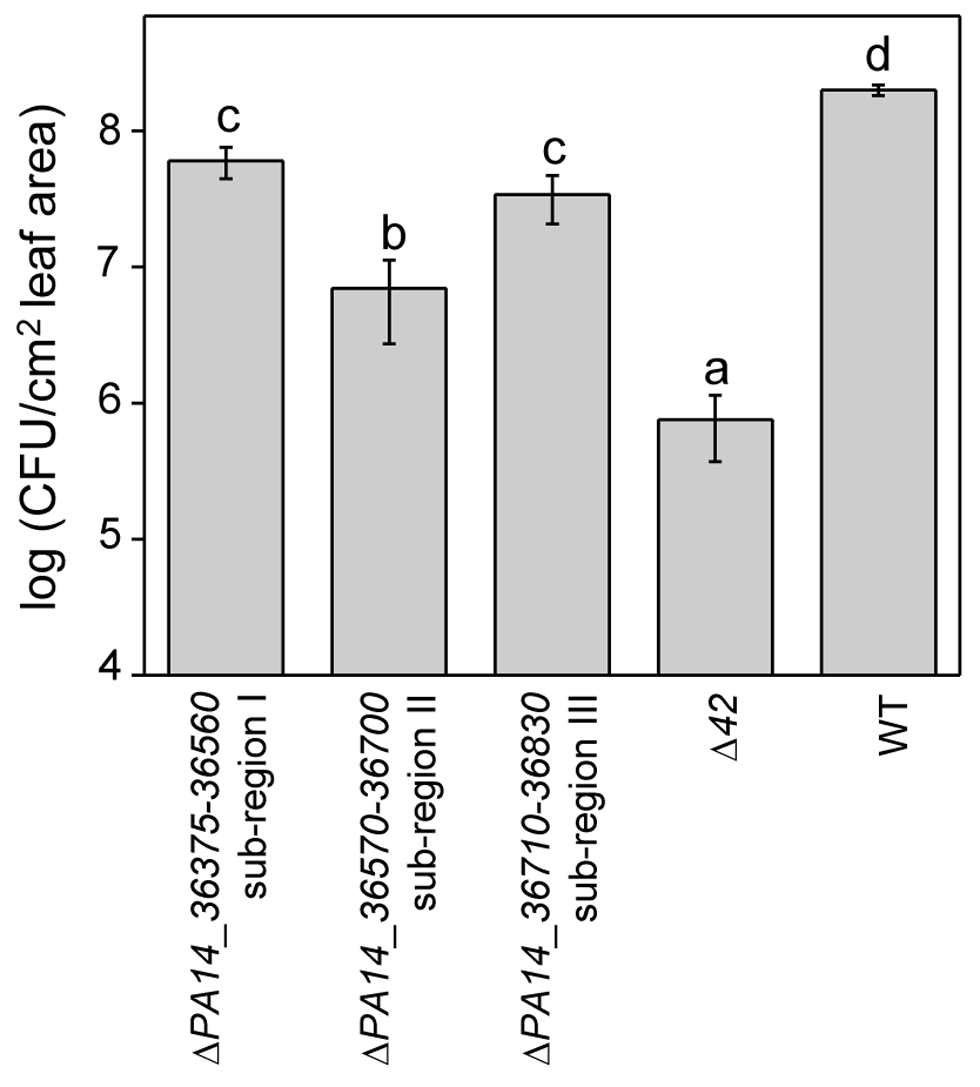

Supplement: Figure S4 — Growth of P. aeruginosa Δ42, three sub-region in-frame deletion mutants, and PA14 wild-type in Arabidopsis Col-0 leaves. Plants were inoculated and incubated as described in Materials and Methods. The leaves were harvested 3 days post-inoculation. Data represent the mean of bacterial titers ± SE of six leaf disks excised from 6 leaves of 3 plants. Different letters above bars denote statistically significant differences (P<0.05, Fisher's PLSD test). See Figure 1 for a description of the mutants. (TIF) [file ppat.1003217.s004.tif]

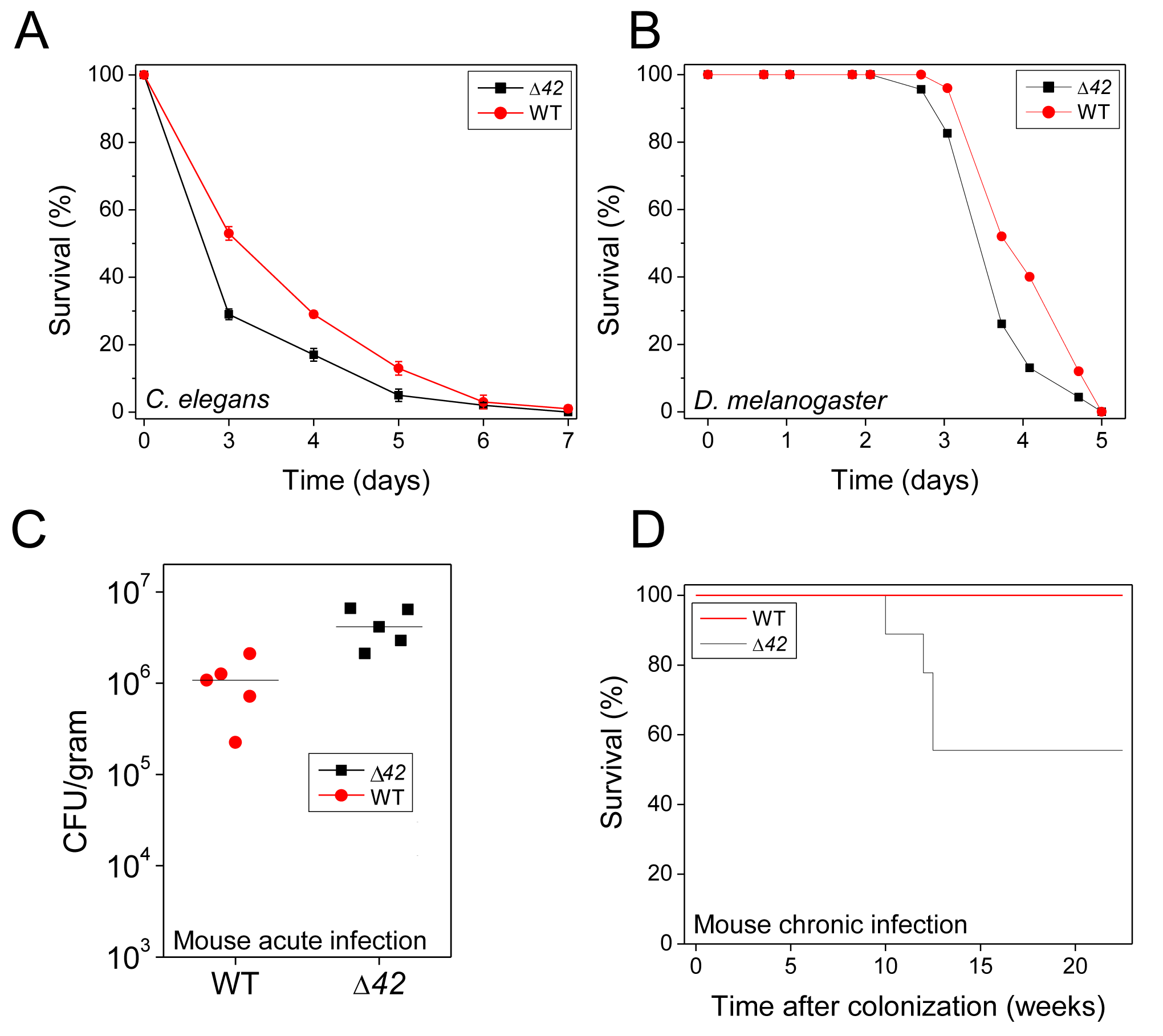

Supplement: Figure S5 — The Δ42 mutant is more virulent than wild-type PA14 in nematodes, insects, and mice. (A) C. elegans are more sensitive to killing by the Δ42 mutant than PA14 wild-type (P<0.004). Mutant fer15;fem1 C. elegans animals were exposed to P. aeruginosa strains and survival was determined as described in Materials and Methods. Data at each time point correspond to the average of three plates per strain, each with approximately 40 animals per plate, and are representative of two independent experiments. (B) D. melanogaster infected with Δ42 die faster than flies infected with PA14 wild-type (P<0.03). D. melanogaster strain Oregon R was infected with P. aeruginosa and approximately 25 flies per vial were scored several times a day for survival throughout the time course of infection. Data are representative of four independent experiments carried out with two different D. melanogaster lines (Oregon R and w[118]). (C) The Δ42 mutant is more virulent in a murine acute lung infection model. See Materials and Methods for details of infection protocol. CFU/gram of lung tissue of mice infected with Δ42 mutant is 3.8-fold higher than with wild-type PA14 18 hours post intranasal infection (P<0.01, Mann-Whitney U test). Data are representative of two independent experiments. (D) FABP-CFTR transgenic mice are more susceptible to killing by Δ42 mutant than by PA14 wild-type after oropharyngeal colonization (P<0.04, log rank test). All mice in both groups (n = 9 for Δ42; n = 8 for PA14 WT) had positive throat cultures for the duration of the experiment after initial colonization by exposure to bacteria in drinking water for one week. (TIF) [file ppat.1003217.s005.tif]

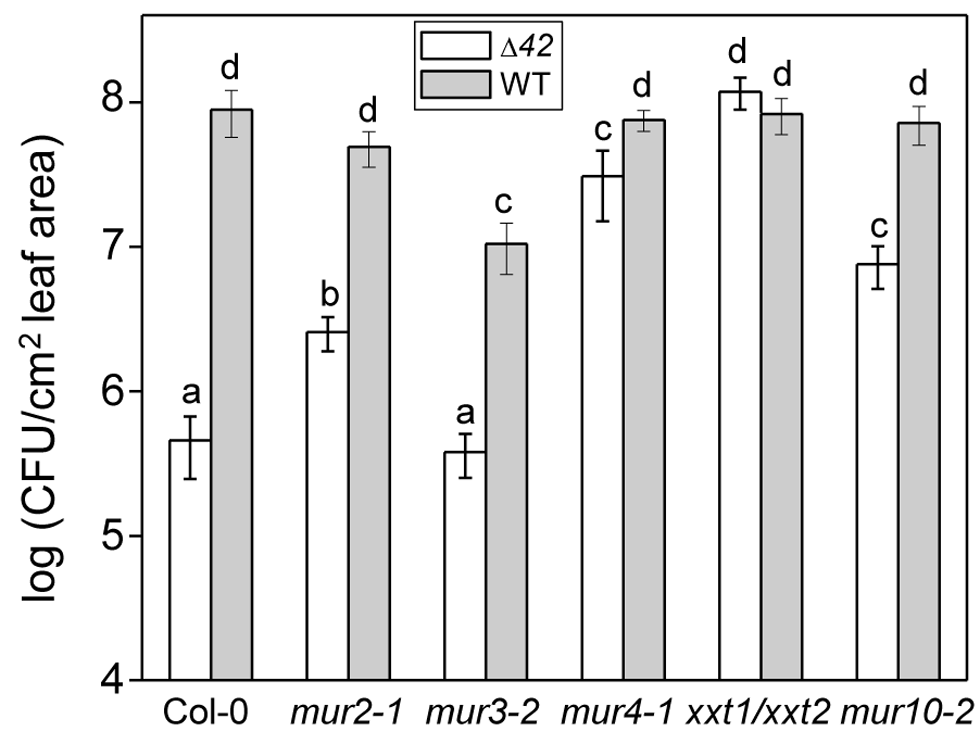

Supplement: Figure S6 — The in planta growth defect of the Δ42 mutant is suppressed by Arabidopsis cell wall mutants. Growth of PA14 wild-type or Δ42 3 days post infiltration in Arabidopsis cell wall mutants mur2-1, mur3-2, mur4-1, mur10-2 and xxt1/xxt2. Data represent the mean of bacterial titers ± SE of six leaf disks excised from 6 leaves of 3 plants. Letters above bars denote statistically significant differences (P<0.05, Fisher's PLSD test). The experiments were repeated at least two times. (TIF) [file ppat.1003217.s006.tif]

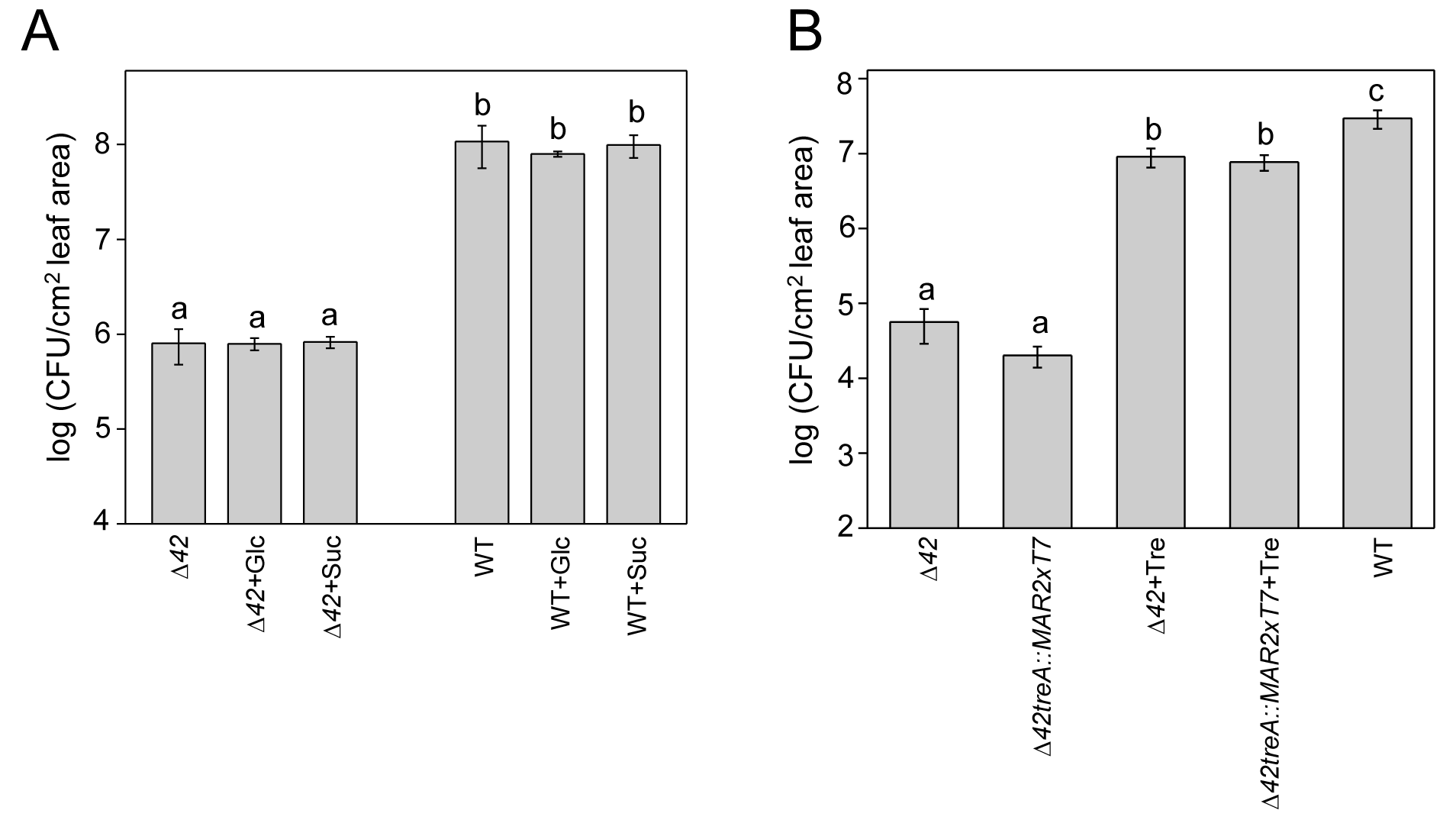

Supplement: Figure S7 — The growth of the Δ42 mutant in planta is not limited by lack of a carbon source. (A) The growth of the Δ42 mutant in planta is not suppressed by 1.25 mg/ml glucose or 2.5 mg/ml sucrose. (B) Suppression of the growth defect of Δ42treA::MAR2xT7 with trehalose in Arabidopsis leaves. Plants were inoculated and incubated as described in Materials and Methods and leaves were harvested 3 days post-inoculation. In (A) and (B), data represent the mean of bacterial titers ± SE of six leaf disks excised from 6 leaves of 3 plants. Different letters above bars denote statistically significant differences (P<0.05, Fisher's PLSD test). The experiments were repeated at least two times. (TIF) [file ppat.1003217.s007.tif]

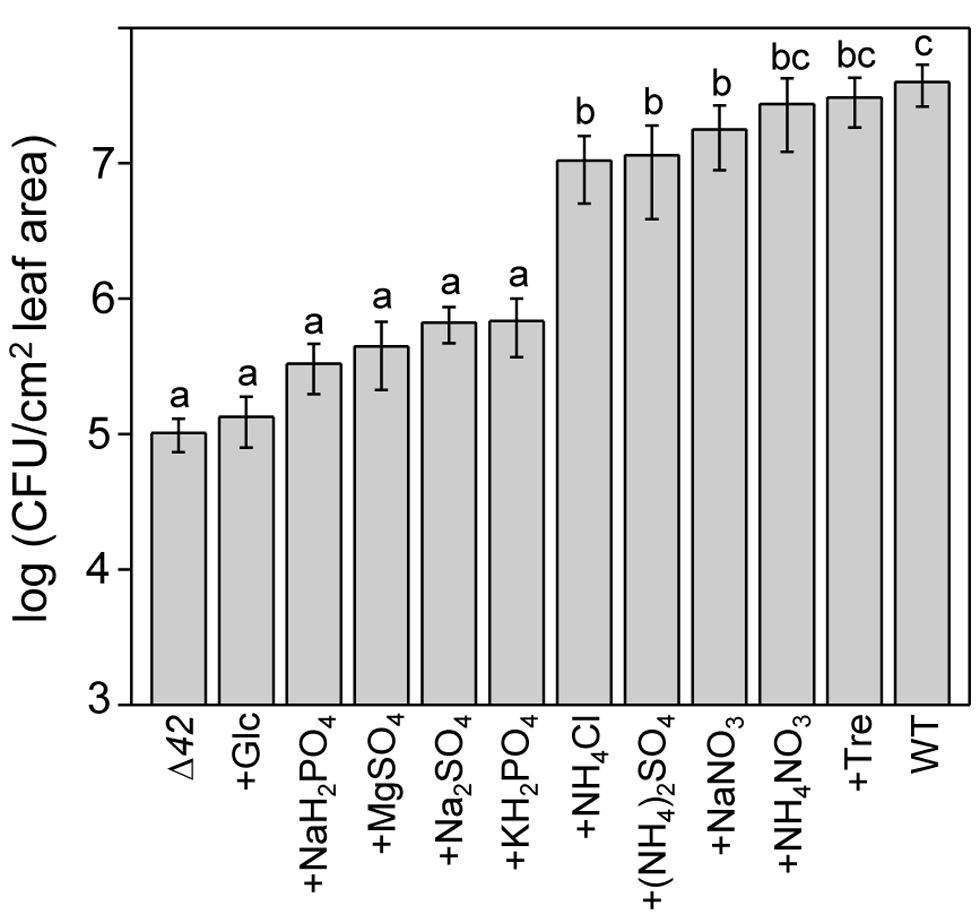

Supplement: Figure S8 — The in planta growth defect of the Δ42 mutant is suppressed by ammonium or nitrate ions. Leaves of four-week-old Arabidopsis Col-0 plants were infiltrated with PA14 wild-type or Δ42 as described in Materials and Methods except that the infiltration solution contained various phosphate, sulfate, nitrate, or ammonium salts at 1 mM. Suppression of the growth defect of Δ42 with 2.5 mg/ml trehalose (Tre) and 1.25 mg/ml glucose (Glc) were tested as positive and negative controls, respectively. Data represent the mean of bacterial titers ± SE of six leaf disks excised from 6 leaves of 3 plants. Letters above bars denote statistically significant differences (P<0.05, Fisher's PLSD test). The experiments were repeated at least two times. (TIF) [file ppat.1003217.s008.tif]

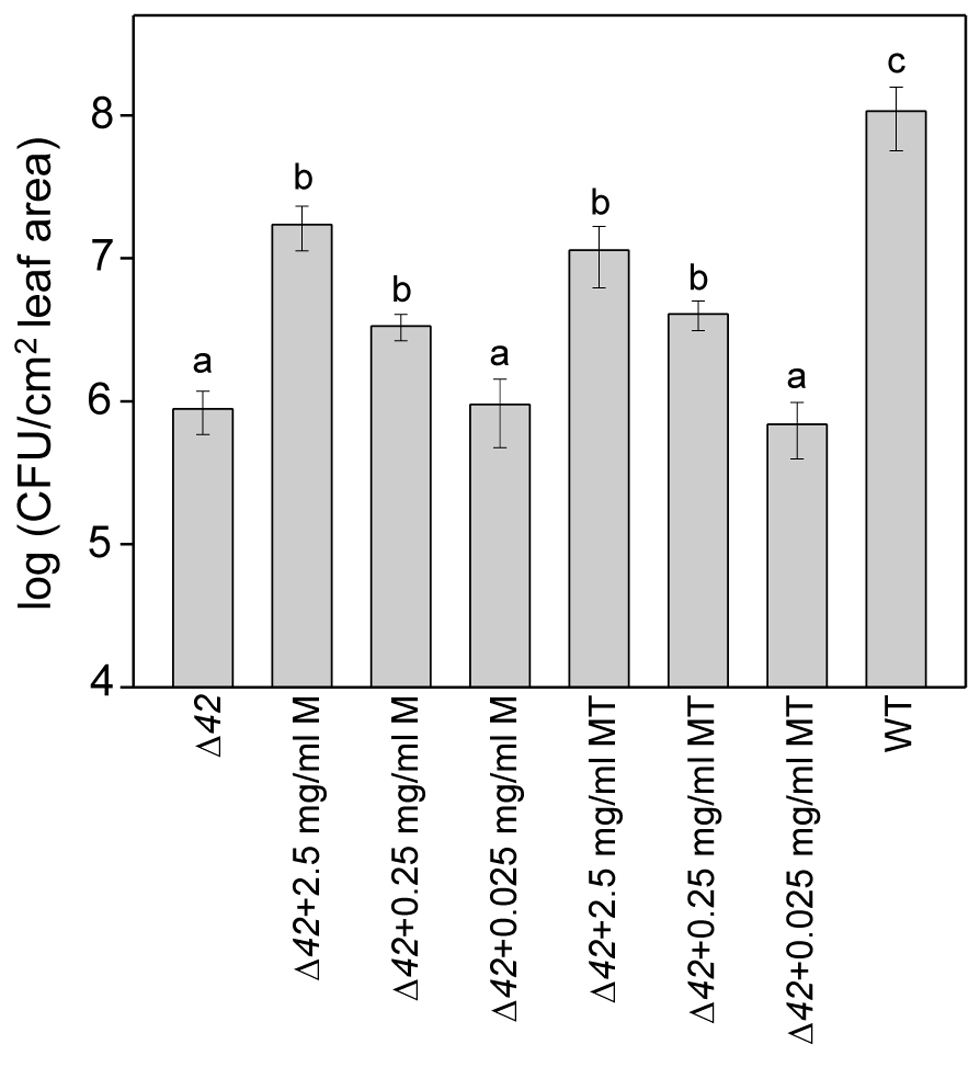

Supplement: Figure S9 — Suppression of attenuation of Δ42 with exogenous maltose and maltotriose. Col-0 plants were infiltrated with PA14 wild-type or Δ42 co-inoculated with maltose (M) or malotriose (MT) at the indicated concentrations. Leaves were harvested 3 days post-infiltration. Data represent the mean of bacterial titers ± SE of six leaf disks excised from 6 leaves of 3 plants. Letters above bars denote statistically significant differences (P<0.05, Fisher's PLSD test). The experiment was repeated at least two times. (TIF) [file ppat.1003217.s009.tif]

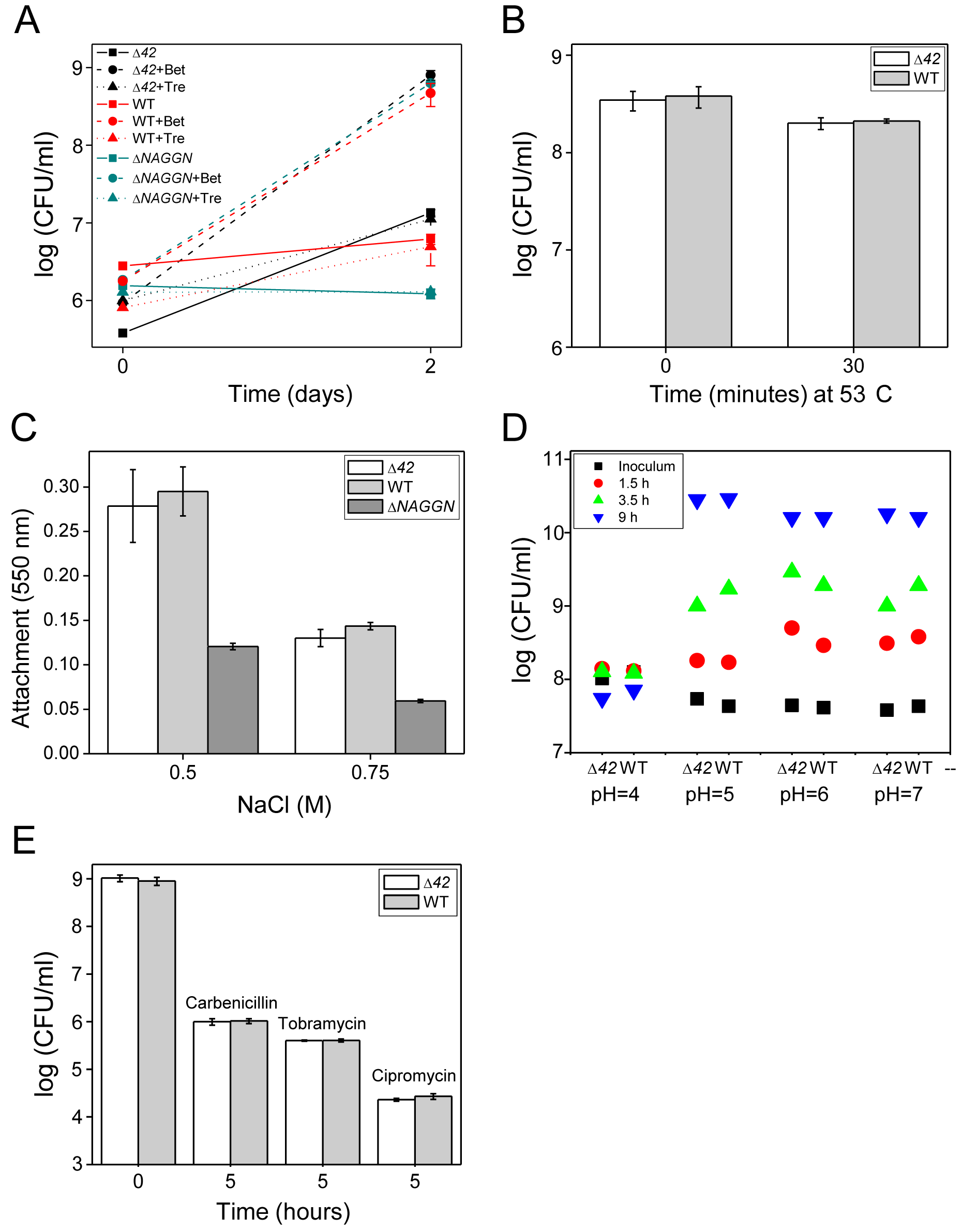

Supplement: Figure S10 — Response of Δ42 to various stress conditions. (A) Rescue of Δ42, ΔNAGGN and PA14 wild-type with betaine but not trehalose under osmotic stress in vitro. Cells were grown at 37°C in MinA medium with 0.5 M NaCl (squares), or with 0.5 M NaCl+1 mM betaine (Bet) (circles) or 1 mM trehalose (Tre) (triangles). Data represent the mean ± SE of 3 replicates. (B) Thermotolerance. Survival of stationary phase bacteria after a 30 minute exposure to 53°C. Temperatures below 53°C were non-lethal and above 56°C were 100% lethal. (C) Biofilm attachment under osmotic stress. Overnight cultures were diluted 1/100 in MinA medium supplemented with 0.5 or 0.75 M NaCl. Attachment assays were performed as described in Materials and Methods. (D) Growth under pH stress. Cultures were grown to stationary phase in LB medium adjusted to pH 4, 5, 6, or 7. (E) Persistence assay. Persisters were determined by exposure of stationary cultures (inoculum, time point zero on x axis) to 6 µg/ml tobramycin, 2 µg/ml ciprofloxacin or 3 mg/ml carbenicillin. The assay was performed as described in Materials and Methods. Based on analysis of variance (ANOVA) and Fisher's PLSD test (P<0.05), there was no significant differences between Δ42 and PA14 wild-type in any of the assays (A–E). (TIF) [file ppat.1003217.s010.tif]
