# Supplementary material for: Trehalose Biosynthesis Promotes Pseudomonas aeruginosa Pathogenicity in Plants
Source: PLoS Pathog. 2013 Mar 7;9(3):e1003217. doi: 10.1371/journal.ppat.1003217 (PMC3591346; doi:10.1371/journal.ppat.1003217)
Supplement: Table S1 — Growth of P. aeruginosa mutants in Arabidopsis Col-0 leaves. Plants were inoculated and incubated as described in Materials and Methods and leaves were harvested 3 days post-inoculation. Data represent the mean of bacterial titers ± SE of six leaf disks excised from 6 leaves of 3 plants. Different superscript letters denote statistically significant differences (P<0.05, Fisher's PLSD test). (DOC) [file ppat.1003217.s011.doc]

| **Supplementary Table 1.** Growth of *P. aeruginosa* mutants in Arabidopsis Col-0 leaves* | | |
| --- | --- | --- |
| Mutants | Predicted Function | CFU/cm^2^ (Fold less than WT) |
| PA14_36390::MAR2xT7 | methyltransferase | 0.8 ± 0.4a |
| PA14_36410::MAR2xT7 | conserved hypothetical protein | 0.6 ± 0.1a |
| PA14_36420::MAR2xT7 | PAS/PAC sensor hybrid histidine kinase | 2.4 ± 0.7a |
| PA14_36500::MAR2xT7 | putative cellulase/peptidase | 3.1 ± 1.4a |
| PA14_36520::MAR2xT7 | HHE hemerythrin cation binding protein | 1.7 ± 0.9a |
| PA14_36550::MAR2xT7 | conserved hypothetical protein | 0.6 ± 0.1a |
| PA14_36570::MAR2xT7 | GlgA, glycogen synthase | 19.7 ± 8.9b |
| PA14_36580::MAR2xT7 | TreZ, maltooligosyltrehalose trehalohydrolase | 15.6 ± 7.0b |
| PA14_36590::MAR2xT7 | MalQ , 4-alpha-glucanotransferase | 2.1 ± 0.7a |
| PA14_36630::MAR2xT7 | glgX, glycogen debranching enzyme | 1.9 ± 1.9a |
| PA14_36670::MAR2xT7 | protein of unknown function DUF72 | 2.6 ± 1.3a |
| PA14_36680::MAR2xT7 | putative metal-dependent hydrolase | 2.1 ± 1.1a |
| PA14_36710::MAR2xT7 | GlgB, 1,4-alpha-glucan branching enzyme | 1.2 ± 0.6a |
| PA14_36730::MAR2xT7 | TreS, putative trehalose synthase | 2.3 ± 0.6a |
| PA14_36760::MAR2xT7 | putative KU domain protein | 2.1 ± 0.9a |
| PA14_36810::MAR2xT7 | KatE, catalase HPII | 0.4 ± 0.1a |
| PA14_23070::MAR2xT7 | Zwf, glucose-6-phosphate 1-dehydrogenase | 2.9 ± 1.3a |
| ∆*PA14_36500* | putative cellulase/peptidase | 3.0 ± 1.3a |
| ∆*PA14_36480-36520* | putative putative cellulase/peptidase operon | 3.8 ± 3.4a |
| ∆*PA14_19350-19370* (∆*NAGGN*) | putative *N*-acetylglutaminylglutamine amide (NAGGN) synthesis operon | 2.3 ± 1.0a |
| *Plants were inoculated and incubated as described in Materials and Methods and leaves were harvested 3 days post-inoculation. Data represent the mean ± SE of six replicate samples. Different superscript letters denote statistically significant differences (P < 0.05, Fisher’s PLSD test). | | |

Table S1
