# Supplementary material for: Trehalose Biosynthesis Promotes Pseudomonas aeruginosa Pathogenicity in Plants
Source: PLoS Pathog. 2013 Mar 7;9(3):e1003217. doi: 10.1371/journal.ppat.1003217 (PMC3591346; doi:10.1371/journal.ppat.1003217)
Supplement: Table S2 — Predicted functions and orthologs of PA14_36375-36830 genes across sequenced Pseudomonas genomes. Putative functions of PA14_36375-36830 genes were assigned as described in Materials and Methods. Genes of the reference taxon PA14 are indicated in the left column (PA14 loci), and different pseudomonad genomes are indicated on the top row. The presence of an ortholog is indicated by a checkmark. Boxes of identical hue indicate that the genes are contiguous in a particular genome, with the lighter shades on the top strand, and darker shades on the bottom strand. Checks in white boxes indicate an ortholog that is not contiguous with other PA14_36375-36830 block orthologs. (PDF) [file ppat.1003217.s012.pdf]

Supplementary Table 2. Predicted functions and orthologs of PA14_36375-36830 genes across sequenced *Pseudomonas* genomes*

PA14 loci	Predicted Function	*Pseudomonas aeruginosa 2192*	*Pseudomonas aeruginosa C3719*	*Pseudomonas aeruginosa PA7*	*Pseudomonas aeruginosa PAO1*	*Pseudomonas aeruginosa UCBPP-PA14*	*Pseudomonas entomophila L48*	*Pseudomonas fluorescens Pf-5*	*Pseudomonas fluorescens Pf0-1*	*Pseudomonas mendocina ymp*	*Pseudomonas putida F1*	*Pseudomonas putida GB-1*	*Pseudomonas putida KT2440*	*Pseudomonas putida W619*	*Pseudomonas stutzeri A1501*	*Pseudomonas syringae pv. phaseolicola 1448A*	*Pseudomonas syringae pv. syringae B728a*	*Pseudomonas syringae pv. tomato str. DC3000*
PA14_36375	ABC transporter, ATPase component	✓	✓	✓	✓	✓	✓	✓	✓	✓	✓	✓	✓	✓	✓	✓	✓	✓
PA14_36390	methyltransferase	✓	✓	✓	✓	✓	✓	✓	✓	✓	✓	✓	✓	✓	✓	✓	✓	✓
PA14_36400	hypothetical protein					✓												
PA14_36410	conserved hypothetical protein	✓	✓	✓	✓	✓												
PA14_36420	PAS/PAC sensor hybrid histidine kinase		✓	✓	✓	✓		✓	✓									✓
PA14_36450	conserved hypothetical protein	✓	✓	✓	✓	✓		✓	✓		✓		✓		✓			
PA14_36460	hypothetical protein	✓	✓	✓	✓	✓												
PA14_36470	hypothetical protein	✓	✓	✓	✓	✓												
PA14_36480	hypothetical protein	✓	✓	✓		✓				✓								
PA14_36490	hypothetical protein	✓	✓	✓	✓	✓				✓								
PA14_36500	putative cellulase/peptidase	✓	✓	✓	✓	✓		✓				✓						
PA14_36520	HHE hemerythrin cation binding protein	✓	✓	✓	✓	✓			✓	✓	✓	✓	✓	✓	✓	✓	✓	✓
PA14_36530	conserved hypothetical protein	✓	✓	✓	✓	✓	✓		✓	✓	✓	✓	✓	✓	✓	✓	✓	✓
PA14_36540	peptidase/esterase	✓	✓	✓	✓	✓		✓	✓	✓	✓	✓	✓	✓	✓			
PA14_36550	conserved hypothetical protein	✓	✓	✓	✓	✓		✓	✓	✓	✓	✓	✓	✓				
PA14_36560	hypothetical protein	✓	✓	✓	✓	✓												
PA14_36570	GlgA, glycogen synthase	✓	✓	✓	✓	✓	✓	✓	✓	✓	✓	✓	✓	✓	✓	✓	✓	✓
PA14_36580	TreZ, maltooligosyltrehalose trehalohydrolase	✓	✓	✓	✓	✓	✓	✓	✓	✓	✓	✓	✓	✓	✓	✓	✓	✓
PA14_36590	MalQ , 4-alpha-glucanotransferase	✓	✓	✓	✓	✓	✓	✓	✓	✓	✓	✓	✓	✓	✓	✓	✓	✓
PA14_36605	TreY, trehalose biomaltooligosyltrehalose synthase	✓	✓	✓	✓	✓	✓	✓	✓	✓	✓	✓	✓	✓	✓	✓	✓	✓
PA14_36620	conserved hypothetical protein	✓	✓	✓	✓	✓	✓	✓	✓	✓	✓	✓	✓	✓				
PA14_36630	glgX, glycogen debranching enzyme	✓	✓	✓	✓	✓	✓	✓	✓	✓	✓	✓	✓	✓	✓	✓	✓	✓
PA14_36650	CBS domain-containing protein	✓	✓	✓	✓	✓				✓								
PA14_36660	threonine dehydrogenasefamily	✓	✓	✓	✓	✓		✓	✓	✓	✓	✓	✓	✓	✓	✓	✓	✓
PA14_36670	protein of unknown function DUF72	✓	✓	✓	✓	✓		✓	✓	✓	✓		✓	✓	✓		✓	
PA14_36680	putative metal-dependent hydrolase	✓	✓	✓	✓	✓	✓	✓	✓	✓	✓	✓	✓	✓	✓	✓	✓	✓
PA14_36690	cardiolipin synthase 2	✓	✓	✓	✓	✓	✓	✓	✓	✓	✓	✓	✓	✓	✓	✓	✓	✓
PA14_36700	transmembrane protein	✓	✓	✓	✓	✓	✓		✓	✓	✓	✓	✓	✓		✓	✓	✓
PA14_36710	GlgB, 1,4-alpha-glucan branching enzyme	✓	✓	✓	✓	✓	✓	✓	✓	✓	✓	✓	✓	✓	✓	✓	✓	✓
PA14_36730	putative trehalose synthase	✓	✓	✓	✓	✓	✓	✓	✓	✓	✓	✓	✓	✓	✓	✓	✓	✓
PA14_36740	putative alpha-amylase family protein	✓	✓	✓	✓	✓	✓	✓	✓	✓	✓	✓	✓	✓	✓	✓	✓	✓
PA14_36760	putative KU domain protein	✓	✓	✓	✓	✓	✓		✓	✓	✓	✓	✓	✓	✓	✓	✓	✓
PA14_36770	hypothetical protein	✓	✓	✓	✓	✓												
PA14_36780	putative Mg(2+) transporter	✓	✓	✓	✓	✓	✓	✓		✓	✓	✓	✓	✓	✓			
PA14_36790	hypothetical protein			✓		✓												
PA14_36810	KatE, catalase HPII	✓	✓	✓	✓	✓		✓	✓	✓	✓	✓	✓	✓	✓	✓	✓	✓
PA14_36820	conserved hypothetical protein	✓	✓	✓	✓	✓				✓	✓	✓			✓			
PA14_36830	hypothetical protein	✓	✓	✓	✓	✓												

*Putative functions of PA14_36375-36830 genes were assigned as described in Methods. Genes of the reference taxon PA14 are indicated in the left column (PA14 loci), and different pseudomonad genomes are indicated on the top row. The presence of an ortholog is indicated by a checkmark. Boxes of identical hue indicate that the genes are contiguous in a particular genome, with the lighter shades on the top strand, and darker shades on the bottom strand. Checks in white boxes indicate an ortholog that is not contiguous with other PA14_36375-36830 block orthologs.
