# Supplementary material for: Trehalose Biosynthesis Promotes Pseudomonas aeruginosa Pathogenicity in Plants
Source: PLoS Pathog. 2013 Mar 7;9(3):e1003217. doi: 10.1371/journal.ppat.1003217 (PMC3591346; doi:10.1371/journal.ppat.1003217)
Supplement: Table S3 — Growth, biofilm formation and motility of Δ42 and PA14 wild-type. Growth rate (h−1) of Pseudomonas strains in minimal media (M63) was calculated by the equation for exponential growth (see Materials and Methods). Biofilm formation was measured as attachment to polyvinylchloride plates in absorbance units (OD550; see Materials and Methods). Swimming and twitching motility are represented as a radius of a halo in cm (see Materials and Methods). Two independent Δ42 deletion constructs were tested. Data represent the mean ± SE. Based on analysis of variance (ANOVA) and Fisher's PLSD test (P<0.05), there was no significant differences between Δ42 mutant and PA14 wild-type in any of the assays. (DOC) [file ppat.1003217.s013.doc]

| **Supplementary Table 3.** Growth, biofilm formation and motility of ∆*42* and PA14 wild-type | | | | | |
| --- | --- | --- | --- | --- | --- |
| Strains | Growth rate M63)^1^ | Biofilm (LB)^2^ | Biofilm (M63)^2^ | Motility (swimming)^3^ | Motility (twitching)^3^ |
| ∆*42*-1 | 1.40 ± 0.04 | 0.39 ± 0.03 | 0.51 ± 0.03 | 0.105 ± 0.003 | 0.05 ± 0.00 |
| ∆*42*-2 | 1.42 ± 0.07 | 0.36 ± 0.02 | 0.50 ± 0.03 | 0.109 ± 0.004 | 0.06 ± 0.01 |
| WT | 1.44 ± 0.07 | 0.39 ± 0.03 | 0.53 ± 0.03 | 0.109 ± 0.007 | 0.06 ± 0.02 |
| ^1^Growth rate (h^-1^) of *Pseudomonas* strains in minimal media (M63) was calculated by the equation for exponential growth (see Methods).  ^2^Biofilm formation was measured as attachment to polyvinylchloride plates in absorbance units (OD550; see Methods).  ^3^Swimming and twitching motility are represented as a radius of a halo in cm (see Methods).  Two independent ∆*42* deletion constructs were tested. Data represent the mean ± SE. Based on analysis of variance (ANOVA) and Fisher’s PLSD test (P < 0.05), there was no significant differences between ∆*42* mutant and PA14 wild-type in any of the assays. | | | | | |

Table S3
